# ﻿Three new species and two new records of the genus *Laccaria* (Agaricales, Basidiomycota) from subtropical China based on morphological and multi-locus phylogenetic evidence

**DOI:** 10.3897/mycokeys.123.156526

**Published:** 2025-10-13

**Authors:** You-Di Xu, Ping Zhang, Zuo-Hong Chen, Zheng-Mi He

**Affiliations:** 1 College of Life Sciences, Hunan Normal University, Changsha 410081, China Hunan Normal University Changsha China

**Keywords:** *
Laccaria
*, morphological characters, phylogenetic analysis, spore ornamentation, taxonomy

## Abstract

*Laccaria* is a large genus within the family Hydnangiaceae, and many potential species remain to be discovered in China. The present study describes three new species, *L. carminostipes*, *L. mangshanensis* and *L. sinolateritia*, and reports two new records, *L. japonica* and *L. versiforma*, which were originally discovered in Japan and Korea, respectively. These species were collected from subtropical mixed forests and identified based on morphological and phylogenetic evidence. Our phylogenetic analysis of the concatenated nucleotide sequences of ITS, LSU, *TEF1* and *RPB2* demonstrated that the three new species each formed a distinct clade, clearly separated from other known *Laccaria* species. A detailed description and illustrations of these species are also provided.

## ﻿Introduction

The genus *Laccaria* Berk. & Broome is a group of ecologically significant ectomycorrhizal fungi> that inhabit soil (Wilson et al. 2016, 2017). The latest DNA-based phylogenetic evidence supports its placement within the family Hydnangiaceae Gäum. & C.W. Dodge, suborder Agaricineae Fries, order Agaricales Underw (Vizzini et al. 2024). The diagnostic characters for *Laccaria* include collybioid to omphaloid basidiomata, vividly orange to brown or purple pileus with thick, sparsely arranged lamellae, and globose to subglobose echinulate basidiospores (Berkeley and Broome 1883; Mueller 1984, 1991a; Mueller and Vellinga 1986). Ecologically, species of *Laccaria* always form symbiotic relationships with trees, such as Pinaceae Lindl., Myrtaceae Juss., Salicaceae Juss., Fagaceae Candolle., Dipterocarpaceae Blume., Nothofagaceae Ørsted. and a part of Fabaceae Lindl. (Mueller1991; Wilson et al. 2017). The importance of this genus lies in its ability to provide nutrients to its plant partners and to engage in nutrient cycling, which is crucial for forest function and stability (Simard 2009; van der Heijden et al. 2015; Wilson et al. 2017). Furthermore, the basidiomata of some *Laccaria* are known to be edible, e.g., *L. alba* Zhu L. Yang & L. Wang, *L. laccata* Cooke, *L. amethystina* Cooke, *L. angustilamella* Zhu L. Yang & L. Wang, *L. aurantia*F. Popa et al, *L. bullipellis* A.W. Wilson & G.M. Muell., and *L. himalayensis* A.W. Wilson & G.M. Muell (Guzmán 2016; Li et al. 2015; Wu et al. 2019; Mao 2020; Wang et al. 2022).

Since *Laccaria* was established by Berkeley and Broome (1883), numerous mycologists have contributed to its taxonomy. To date, ca. 120 species have been reported worldwide (Berkeley and Broome 1883; Singer 1967; Besson and Kühner 1971; Mueller and Sundberg 1981; Osmundson et al. 2005; Popa et al. 2016; Campi et al. 2017; Ramos et al. 2017; Corrales et al. 2020; Cui et al. 2021; Dovana et al. 2021), with the majority discovered in Europe, North America. Recently, the number of *Laccaria* species recognized in Asia is increasing, driven by growing awareness of the ecological and economic importance of this genus (Cho et al. 2018, 2020; Deepna Latha et al. 2019; Tang et al. 2024; Thapa et al. 2024). In China, 25 new species of *Laccaria* have been described since the year 2000 (Wang et al. 2004; Wilson et al. 2013; Popa et al. 2014; Luo et al. 2016; Vincenot et al. 2017; Li 2020; Cui et al. 2021; Wang et al. 2022; Zhang et al. 2023; Li et al. 2024). They were found in Southern China, a region whose biodiversity is reflected in its diverse forest ecosystems. Furthermore, most of them were discovered from Yunnan Province. However, studies on the diversity of *Laccaria* taxa in other provinces of Southern China remain limited. In this study, (i) two new species from Hunan Province and one new species from Yunnan Province are proposed based on both molecular and morphological evidence; (ii) Chinese specimens of *L. japonica* Popa & K. Nara and *L. versiforma* H.J. Cho & Y.W. Lim, collected from Guizhou, Hubei and Hunan Provinces, are reported for the first time.

## ﻿Materials and methods

### ﻿Specimen collection

A total of 28 specimens of *Laccaria* were involved in this study. These specimens were collected from multiple locations in Southern China, including Yunnan, Guizhou, Hubei, Jiangxi, and Hunan Provinces, between 2016 and 2024. After dehydration using heat or silica gel, they were deposited in the Mycological Herbarium of Hunan Normal University (MHHNU, Changsha, China). Information regarding the specimens, including species name, GenBank accession number, voucher and location, is provided in Table 1.

**Table 1.** Sequences used for four-locus phylogenetic analysis and their corresponding. GenBank accession numbers. Species and accession numbers in bold indicate a newly generated sequence in this study.

Species	voucher	locality	ITS	28S	*tef1*	*Rpb2*	References
*Laccaria acanthospora* (T)	AWW485	Tibet, China	JX504102	JX504186	KU686073	KU685916	Wilson et al. (2013)
*L. acanthospora*	MHHNU 12061	Yunnan, China	PV300418	PV300502	PV339949	PV467460	This study
*L. acanthospora*	HKAS45998	Tibet, China	KU685719	KU685870		KU686069	Wilson et al. (2013)
*L. alba*	AWW438	Yunnan, China	JX504094	JX504178	KU686072	KU685912	Wilson et al. (2013)
*L. alba*	F1121461	China	JX504129	JX504209			Wilson et al. (2013)
*L. alba*	TPML20120807-69	Korea	MG519542	MG519583	MG551649	MG551616	Cho et al. (2018)
*L. alba*	MHHNU 32470	Hunan, China	PV300419			PV467461	This study
*L. alba*	MHHNU 20007	Guizhou, China	PV300420	PV300503		PV467462	This study
*L. ambigua* (T)	PDD89696*	New Zealand	KU685725	KU685876	KU686132	KU686018	Wilson et al. (2016)
*L. amethysteo-occidentalis*	AWW556	USA	JX504107	JX504191		KU685919	Wilson et al. (2013)
*L. amethysteo-occidentalis*	AWW590	USA	JX504112	JX504195		KU685923	Wilson et al. (2013)
*L. amethystina*	GMM7633	France	JX504154	AF440665			Wilson et al. (2013)
*L. amethystina*	GMM7041	Russia	KU685654	KU685797		KU685940	Wilson et al. (2016)
*L. amethystina*	KHLA06002	USA	KU685759	KU685910	KU686162	KU686059	Wilson et al. (2016)
*L. amethystina*	GMM7621	France	JX504150	JX504224	KU686152	KU686046	Popa et al. (2014)
*L. angustilamella*	HKAS58714	Yunnan, China	JX504168	JX504244			Wilson et al. (2013)
*L. araneosa*	KNU20120912-25	Korea	MG519550	MG519590	MG551656	MG551623	Cho et al. (2018)
*L. araneosa*	MHHNU 34707	Hunan, China	PV300421	PV300504	PV339950	PV467463	This study
*L. araneosa*	MHHNU 34708	Hunan, China	PV300422	PV300505	PV339951	PV467464	This study
L. araneosa (T)	KNU20120912-40	Korea	MG519548	MG519588	MG551654	MG551621	Cho et al. (2018)
*L. aurantia*	MB-FB-001109	Yunnan, China	JQ681209				Popa et al. (2014)
*L. aurantia*	GMM6172	Yunnan, China	KU685645	KU685789		KU685931	Wilson et al. (2016)
*L. aurantia*	MHHNU 34709	Hubei, China	PV300423	PV300506	PV339952	PV467465	This study
*L. aurantia*	MHHNU 11885	Guizhou, China	PV300424				This study
*L. aurantia* (T)	KUN-F 78557	Yunnan, China	JQ670895				Popa et al. (2014)
*L. aurantiaca*	KUN-HKAS123246	Yunnan, China	PQ651573	PQ720998	PQ753350	PQ753336	Tang et al. (2025)
*L. aurantiaca* (T)	KUN-HKAS123244	Yunnan, China	PQ651572	PQ720997	PQ753349	PQ753335	Tang et al. (2025)
*L. bicolor*	KA130253	Korea	MG519524	MG519570	MG551636	MG551599	Cho et al. (2018)
*L. bicolor*	AWW537	USA	JX504105	JX504189			Wilson et al. (2013)
*L. bicolor*	GMM7620	France	JX504149	JX504223			Wilson et al. (2013)
*L. bicolor*	HKAS44062	Yunnan, China	JX504159	JX504235			Wilson et al. (2013)
*L. bicolor*	F1121424	China	JX504127	JX504207		KU686064	Wilson et al. (2013)
*L. bicolor*	GMM6131	China	JX504131	JX504210	KU686079	KU685930	Wilson et al. (2013)
*L. bicolor*	AWW596	USA	JX504116	JX504199			Wilson et al. (2013)
*L. bicolor*	GMM6094	China	KM067831	KU685788		KU686067	Wilson et al. (2016)
*L. bicolor*	GMM2692	Chile	KU685630	KU685774			Wilson et al. (2016)
*L. bicolor*	A0103	Japan	JN942778	JN939731			Direct Submission
*L. bicolor*	MHHNU 11595	Jiangxi, China	PV300425	PV300507	PV339953	PV467466	This study
L. aff. bicolor	AWW539	USA	KM067817	KU685763			Wilson et al. (2016)
*L. brunnea*	KUN-HKAS 123243	Yunnan, China	PQ651575	PQ721004	PQ753352	PQ753338	Tang et al. (2025)
*L. brunnea*	MHHNU 9692	Guizhou, China	PV300439	PV300520	PV339963	PV467477	This study
*L. brunnea* (T)	KUN-HKAS 123286	Yunnan, China	PQ651574	PQ721003	PQ753351	PQ753337	Tang et al. (2025)
*L. bullipellis* (T)	AWW465	Tibet, China	JX504100	JX504184		KU685914	Wilson et al. (2013)
*L. canaliculata*	GMM7209	Australia	JX504136	JX504212		KU685944	Wilson et al. (2013)
*L. canaliculata*	GMM7227	Australia	KU685666	KU685809	KU686089	KU685952	Wilson et al. (2016)
*L. carminostipes*	MHHNU 31552	Hunan, China	PV300440	PV300521			This study
*L. carminostipes*	MHHNU 34706	Hunan, China	PV300442		PV339965	PV467479	This study
*L. carminostipes*	MHHNU 11944	Yunnan, China	PV300443	PV300522	PV339966	PV467480	This study
*L. carminostipes* (T)	MHHNU 31553	Hunan, China	PV300441		PV339964	PV467478	This study
*L. cflaccata*	A3394	Japan	JN942788	JN939770		JN993522	Direct Submission
*L. cflaccata*	A2987	Japan	JN942786	JN939739		JN993521	Direct Submission
*L. cflaccata*	AWW555	USA		KU685764	KU686074	KU685918	Wilson et al. (2016)
*L. cinnabarina*	KUN-HKAS83381	Yunnan, China	OR722588	OR722601	PP171545	PP171558	Li et al. (2024)
*L. cinnabarina* (T)	KUN-HKAS80885	Yunnan, China	OR722587	OR722595			Li et al. (2024)
*L. darjeelingensis* (T)	CUHAM788	INDIA	OQ607624				Thapa et al. (2024)
*L. fagacicola*	KUN-HKAS107731	Yunnan, China	MW540807	OR722594	PP171550	PP171554	Cui et al. (2021)
*L. fagacicola*	MHHNU 11978	Yunnan, China	PV300426	PV300508	PV339954	PV467467	This study
*L. fagacicola* (T)	KUN-HKAS90435	Yunnan, China	MW540806	OR122593	PP171549		Cui et al. (2021)
*L. fengkaiensis*	KUN-HKAS106741	Guangdong, China	MN585658				Li (2020)
*L. fengkaiensis* (T)	KUN-HKAS106739	Guangdong, China	MN585657	MN621238			Li (2020)
*L. fibrillosa*	GMM7508	New Zealand	KU685706	KU685847		KU685989	Wilson et al. (2016)
*L. fulvogrisea* (T)	KUN-F78556	Yunnan, China	JQ670896				Popa et al. (2014)
*L. galerinoides*	F1081213	Chile	KU685634	KU685778	KU686078	KU685929	Wilson et al. (2016)
*L. galerinoides*	F1080983	Argentina	KU685632	KU685776	KU686077	KU685927	Wilson et al. (2016)
*L. glabripes*	GMM7521	New Zealand	KU685708	KU685849	KU686117	KU685991	Wilson et al. (2016)
*L. glabripes*	GMM7534	New Zealand	KU685711	KU685852			Wilson et al. (2016)
*L. gomezii*	F1104722	Costa Rica	KU685639	KU685782			Wilson et al. (2016)
*L. guizhouensis*	HMAS352266	Guizhou, China	OP244891				Zhang et al. (2024)
*L. guizhouensis* (T)	HMAS352265	Guizhou, China	OP244890				Zhang et al. (2024)
*L. himalayensis*	AWW463	Tibet, China	JX504098	JX504182		KU685913	Wilson et al. (2013)
*L. himalayensis* (T)	AWW484	Tibet, China	JX504101	JX504185		KU685915	Wilson et al. (2013)
*L. infundibuliformis* (T)	CUHAM786	INDIA	OQ607560				Thapa et al. (2024)
*L. japonica*	SFC20110921-34	Korea	MG519519	MG519568		MG551596	Cho et al. (2018)
*L. japonica*	HMHHNU 9589	Guizhou, China	PV300427	PV300509			This study
*L. japonica*	MHHNU 34710	Hubei, China	PV300428	PV300510			This study
*L. japonica*	MHHNU 34711	Hunan, China	PV300429	PV300511			This study
*L. japonica* (T)	TNS-F64167	Japan	KU962988				Vincenot et al. (2017)
*L. laccata*	GMM7615	France	JX504148	JX504222			Wilson et al. (2013)
*L. laccata*	SB2067	Portugal	JX504171	JX504248		KU686026	Wilson et al. (2013)
*L. laccata*	SB2133	Portugal	KM067887	KU685884	KU686139	KU686027	Wilson et al. (2016)
*L. laccata*	SB2210	Portugal	KM067890	KU685885			Wilson et al. (2016)
*L. laccata*	GMM7586	Russia	KM067835	KU685859		KU686000	Wilson et al. (2016)
L. laccata var. pallidifolia	Cripps1603	USA	DQ149851				Osmundson et al. (2005)
L. laccata var. pallidifolia	GMM7605	France	KM067844	KU685901	KU686154	KU686048	Wilson et al. (2016)
*L. lateritia*	GMM7220	Australia	KU685662	KU685805		KU685948	Wilson et al. (2016)
*L. lilacina*	GMM7531	New Zealand	KU685709	KU685850	KU686118	KU685992	Wilson et al. (2016)
*L. longipes*	F1092175	USA	KU685637	KU685780			Wilson et al. (2016)
*L. longistriata*	KUN-HKAS123799	Yunnan, China	OQ396727	OR345239	OR347684	OR347686	Li et al. (2024)
*L. longistriata* (T)	KUN-HKAS123801	Yunnan, China	OQ396730		OR347685		Li et al. (2024)
*L. macrocystidia*	GMM7612	France	KM067847	KU685861	KU686122	KU686002	Wilson et al. (2016)
*L. macrocystidia*	GMM7626	France	KM067856	KU685865	KU686125	KU686006	Wilson et al. (2016)
*L. major*	GMM6019	Costa Rica	KU685757	KU685908	KU686160	KU686056	Wilson et al. (2016)
*L. mangshanensis*	MHHNU 8856	Hunan, China	PV300438	PV300519	PV339962	PV467476	This study
*L. mangshanensis* (T)	MHHNU 8850	Hunan, China	PV300437	PV300518	PV339961	PV467475	This study
*L. masoniae*	GMM7473	New Zealand	KU685703	KU685845	KU686116	KU685987	Wilson et al. (2016)
*L. miniata* (T)	GDGM76043	China	OR689440	OR785476			Zhang et al. (2023)
*L. montana*	TWO591(MONT)	-	DQ149865				Osmundson et al. (2005)
*L. montana*	TWO319(MONT)	North America	DQ149862				Osmundson et al. (2005)
L. aff. montana	AWW446	Tibet, China	JX504097	JX504181	KU686157	KU686054	Wilson et al. (2013)
L. aff. montana	GMM7630tibet	Tibet, China	JX504151	JX504225	KU686128	KU686009	Wilson et al. (2013)
*L. moshuijun*	MHHNU 32931	Yunnan, China	PV300430	PV300512	PV339955	PV467468	This study
*L. moshuijun* (T)	KUN-HKAS93732	Yunnan, China	KU962989				Vincenot et al. (2017)
*L. murina*	MHHNU 10903	Hunan, China	PV300431	PV300513		PV467469	This study
*L. murina*	ASIS2021	Korea	MG519554				Cho et al. (2018)
*L. murina*	ASIS24249	Korea	MG519552	MG519592	MG551658	MG551625	Cho et al. (2018)
*L. nanlingensis*	GDGM84949	China	OR689441	OR785477	OR826274	OR835198	Zhang et al. (2023)
*L. nanlingensis* (T)	GDGM84954	China	OR689442	OR785478	OR826273	OR835199	Zhang et al. (2023)
*L. negrimarginata*	GMM7631tibet	Tibet, China	JX504153	JX504227	KU686130	KU686011	Wilson et al. (2013)
*L. negrimarginata* (T)	BAP360	Tibet, China	JX504120				Wilson et al. (2013)
*L. neovinaceoavellanea*	GDGM53063	Jiangxi, China	OR689448	OR785480			Zhang et al. (2023)
*L. neovinaceoavellanea* (T)	GDGM52852	Jiangxi, China	OR689447	OR785479			Zhang et al. (2023)
*L. nobilis*	F1120629	China	JX504124	JX504204			Wilson et al. (2013)
*L. nobilis*	AWW584	USA	JX504110	JX504193		KU685922	Wilson et al. (2013)
*L. oblongospora*	ObiFr	France	GQ406466				Vincenot et al. (2017)
*L. ochropurpurea*	PRL3777	USA	JX504169	JX504246		KU686024	Wilson et al. (2013)
*L. ochropurpurea*	PRL4777	USA	KU685733	KU685883		KU686025	Wilson et al. (2016)
*L. ochropurpurea*	AFTOL447	-		AY700200		DQ472731	Wilson et al. (2016)
*L. ohiensis*	AWW545	USA	JX504106	JX504190		KU685917	Wilson et al. (2016)
*L. ohiensis*	GMM7539	New Zealand	KU685712	KU685853	KU686119	KU685994	Wilson et al. (2016)
*L. ohiensis*	KH_07192006_1	USA	KU685720	KU685871		KU686014	Wilson et al. (2016)
*L. pallidorosea*	HKAS53170	Yunnan, China	MW540809	OR722602	PP171548	PP171555	Cui et al. (2021)
*L. pallidorosea* (T)	HKAS107730	Yunnan, China	MW540808				Cui et al. (2021)
*L. pallidus* (T)	CUHAM787	INDIA	OQ607623				Thapa et al. (2024)
*L. parva* (T)	SFC20120919-40	Korea	MG519525				Cho et al. (2018)
*L. prava*	KUN-HKAS106745	Guangdong, China	MN585661				Li (2020)
*L. prava* (T)	KUN-HKAS106742	Guangdong, China	MN585660				Li (2020)
*L. “proxima*”	GMM7628	France	KM067857	KU685867	KU686127	KU686008	Wilson et al. (2013)
*L. proxima*	GMM7584	Russia	KU685717	KU685858	KU686120	KU685999	Wilson et al. (2016)
*L. proxima*	F1133825	USA	KU685642	KU685786		KU686065	Wilson et al. (2016)
*L. proximella*	F1081079	Argentina	KU685633	KU685777		KU685928	Wilson et al. (2016)
*L. pseudoalba*	HKAS-110664	Thailand	ON557376	ON556491	ON598894	ON598887	Thapa et al. (2024)
*L. pseudoalba* (T)	MFLU-22-0106	Thailand	ON557377	ON556492		ON598886	Thapa et al. (2024)
*L. pseudomontana* (T)	pse1625	USA	DQ149871				Osmundson et al. (2005)
*L. pumila*	GMM7637	France	JX504156	JX504229	KU686158		Wilson et al. (2013)
*L. pumila*	pum1252	North America	DQ149864				Osmundson et al. (2005)
*L. roseoalbescens* (T)	LM5099	Mexico	KJ874328	KJ874331			Montoya et al. (2015)
*L. ruber*	KUN-HKAS123292	Yunnan, China	PQ651571	PQ776318	PQ753348	PQ753334	Tang et al. (2025)
*L. ruber* (T)	KUN-HKAS123291	Yunnan, China	PQ651570	PQ776317	PQ753347	PQ753333	Tang et al. (2025)
*L. rubroalba*	HKAS90758	Yunnan, China	KX449357				Luo et al. (2016)
*L. rubroalba*	MHHNU 11941	Yunnan, China	PV300432	PV300514	PV339956	PV467470	This study
*L. rubroalba* (T)	HKAS90753	Yunnan, China	KX449358				Luo et al. (2016)
*L. rufobrunnea*	GDGM89627	Yunnan, China	OR689444	OR785483			Zhang et al. (2023)
*L. rufobrunnea* (T)	GDGM82878	Yunnan, China	OR689443	OR785482	OR826272	OR835197	Zhang et al. (2023)
*L. salmonicolor*	GMM7602tibet	Tibet, China	JX504145	JX504220			Wilson et al. (2013)
*L. salmonicolor* (T)	GMM7596tibet	Tibet, China	JX504143	JX504218	KU686151	KU686045	Wilson et al. (2013)
*L. sinolateritia*	MHHNU 11958	Yunnan, China	PV300445	PV300524	PV339968	PV467482	This study
*L. sinolateritia* (T)	MHHNU 11956	Yunnan, China	PV300444	PV300523	PV339967	PV467481	This study
*L.* sp 1.	AWW591	USA		KU685769		KU685924	Wilson et al. (2016)
*L.* sp 2.	GMM6012	Costa Rica	KU685758	KU685909		KU686057	Wilson et al. (2016)
*L.* sp 3.	GMM6800	Guatemala	KU685756	KU685907	KU686159	KU686055	Wilson et al. (2016)
*L.* sp 4.	ALB183	China: Tibet	JX504092	JX504176	KU686161	KU686058	Wilson et al. (2013)
*L.* sp 5.	F1123822	USA	KU685760	KU685911		KU686071	Wilson et al. (2016)
*L.* sp 6.	AWW569	USA	JX504108	KU685766		KU685920	Wilson et al. (2013)
*L.* sp 7.	GMM7627	France		KU685866	KU686126	KU686007	Wilson et al. (2016)
*L.* sp 8.	GMM7020	Russia	KU685652	KU685795		KU685938	Wilson et al. (2016)
*L.* sp 9.	GMM6585	Costa Rica	KU685647	KU685791			Wilson et al. (2016)
*L.* sp 10.	T173	China	MT500512	MT500551			Direct Submission
*L.* sp 11.	H160	China	MT500501	MT500542			Direct Submission
*L.* sp 12.	A1800	Taiwan, China	KU685622				Wilson et al. (2016)
*L.* sp 13.	T44	China	MT500502				Direct Submission
*L.* sp 14.	T110	China	MT500508	MT500547			Direct Submission
*L.* sp 15.	GMM6679	Yunnan, China	KU685649	KU685792	KU686081	KU685935	Wilson et al. (2016)
*L.* sp 16.	TWO1166	Thailand	KU685744	KU685895		KU686041	Wilson et al. (2016)
*L.* sp 17.	DED7426	Thailand	KU685628	KU685771	KU686076	KU685926	Wilson et al. (2016)
*L.* sp 18.	TWO1168	Thailand	KU685745	KU685896	KU686146	KU686042	Wilson et al. (2016)
*L.* sp 19.	TWO1150	Thailand	KU685743	KU685894		KU686040	Wilson et al. (2016)
*L.* sp 20.	TWO1178	Thailand	KU685746	KU685897	KU686147	KU686043	Wilson et al. (2016)
*L.* sp 21.	ZT9196	Indonesia	KU685750	KU685900		KU686070	Wilson et al. (2016)
*L.* sp 22.	GMM6776	Yunnan, China	KU685651	KU685794			Wilson et al. (2016)
*L.* sp 23.	HKAS-83382	China	PP191171	PP191170			Direct Submission
*L.* sp 24.	T107	China	MT500506	MT500545			Direct Submission
*L.* sp 25.	T168	China	MT500511	MT500550			Direct Submission
*L.* sp 26.	TWO1184	Thailand	KU685747	KU685898	KU686148		Wilson et al. (2016)
*L.* sp 27.	TWO1194	Thailand	KU685748	KU685899	KU686149		Wilson et al. (2016)
*L.* sp 28.	GMM6583	China	KU685646	KU685790	KU686080	KU685932	Wilson et al. (2016)
*L. spinulosa*	KUN-HKAS122272	Yunnan, China	OR722592	OR722596	PP171552		Li et al. (2024)
*L. spinulosa* (T)	KUN-HKAS129615	Yunnan, China	OR722591	OR722598	PP171551		Li et al. (2024)
*L. squarrosa*	DM93	Mexico	MF669959	MF669966			Ramos et al. (2017)
*L. squarrosa* (T)	DM63	Mexico	MF669958	MF669965			Ramos et al. (2017)
*L. stellata*	MB-002397	Panama	KP877339				Popa et al. (2016)
*L. stellata* (T)	MB-002396	Panama	KP877340				Popa et al. (2016)
*L. stipalba*	MHHNU 11315	Yunnan, China	PV300434	PV300515	PV339958	PV467472	This study
*L. stipalba*	MHHNU 11324	Yunnan, China	PV300435	PV300516	PV339959	PV467473	This study
*L. stipalba*	MHHNU 12057	Yunnan, China	PV300436	PV300517	PV339960	PV467474	This study
*L. stipalba*	KUN-HKAS123285	Yunnan, China	PQ651566	PQ753314	PQ753343	PQ753329	Tang et al. (2025)
*L. stipalba* (T)	KUN-HKAS123300	Yunnan, China	PQ651565	PQ753313	PQ753342	PQ753328	Tang et al. (2025)
*L. subroseoalbescens*	MFLU23-0340	Thailand	PP785398	PP789599			Tang et al. (2024)
*L. subroseoalbescens* (T)	MFLU23-0339	Thailand	PP785397	PP789598			Tang et al. (2024)
*L. tetraspora*	F1080957	Germany	KU685631	KU685775			Wilson et al. (2016)
*L. torosa* (T)	SFC2015090217	Korea	MG519561	MG519598	MG551664	MG551631	Cho et al. (2018)
*L. tortilis*	GMM7635	France	KM067859	KU685906	KU686156	KU686053	Wilson et al. (2016)
*L. tortilis*	F1116205	USA	KU685641	KU685785			Wilson et al. (2016)
*L. tortilis* (T)	ASIS22273	Korea	MG519533	MG519576	MG551644	MG551608	Cho et al. (2018)
*L. trichodermophora*	GMM7733	USA	JX504157	JX504230		KU686013	Wilson et al. (2013)
*L. trichodermophora*	F1111951	Costa Rica	KU685640	KU685784		KU686063	Wilson et al. (2016)
*L. trichodermophora* (T)	TENN42523	USA	DQ149868				Osmundson et al. (2005)
*L. trullisata*	PRL7587	China	JX504170	JX504247	KU686153	KU686047	Wilson et al. (2013)
*L. trullisata*	WCG2072	-	KU685749		KU686150	KU686044	Wilson et al. (2016)
*L. umbilicate*	GDGM82883	China	OR689445	OR785485	OR826270	OR835194	Zhang et al. (2023)
*L. umbilicate* (T)	GDGM82911	China	OR689446	OR785486	OR826268	OR835192	Zhang et al. (2023)
*L. versiforma*	SFC20121010-51	Korea	MG519555	MG519593			Cho et al. (2018)
*L. versiforma*	TPML20121008-03	Korea	MG519560	MG519597	MG551663	MG551630	Cho et al. (2018)
*L. versiforma*	ASIA20939	Korea	MG519557	MG519595	MG551661	MG551628	Cho et al. (2018)
*L. versiforma*	MHHNU 10896	Hunan, China	PV300433		PV339957	PV467471	This study
*L. versiforma* (T)	SFC20120926-01	Korea	MG519556	MG519594	MG551660	MG551627	Cho et al. (2018)
*L. vinaceoavellanea*	A0559	Japan	JN942803	JN939756		JN993512	Direct Submission
*L. vinaceoavellanea*	SFC20150810-10	Korea	MG519539	MG519580	MG551614	MG551646	Cho et al. (2018)
*L. vinaceobrunea*	KH_LA06_018	USA		KU685873		KU686015	Wilson et al. (2016)
*L. violaceonigra*	GMM7580	New Zealand	KU685716	KU685857		KU685998	Wilson et al. (2016)
*L. violaceonigra*	GMM7520	New Zealand	KU685707	KU685848		KU685990	Wilson et al. (2016)
*L. violaceotincta* (T)	CAL1389	India	MK141034				Deepna et al. (2019)
*L. yunnanensis*	HKAS-110636	Thailand	ON557373	ON556487	ON598891	ON598889	Popa et al. (2014)
*L. yunnanensis* (T)	KUN-F78558	Yunnan, China	JQ670897				Popa et al. (2014)
*Mythicomyces corneipes*	AFTOL972	Germany	DQ404393	AY745707	DQ029197	DQ408110	DirectSubmission
*Mythicomyces corneipes*	ES11.10.2. A	Germany	KC964108				DirectSubmission

“T” represents the type specimen of the species.

### ﻿Morphological study

Macroscopic characters of species were described based on field notes and digital images. The size of basidiomata, as determined by pileus width, was described as tiny (<1.5 cm), small (1.5–3 cm), medium-sized (3–5 cm) or large (>5 cm). The color codes mentioned in descriptions are from Kornerup and Wanscher (1978). For microscopic studies, hand-made sections of dried basidiomata were prepared under a stereomicroscope to ensure precise dissection of specific tissues. Microscopic examinations were then conducted using a light microscope. The dried specimens mounted in either a 5% KOH solution or distilled water, with Congo red staining when necessary. Melzer’s reagent was used to test the amyloidity of basidiospores. With preheating, Cotton blue reagent was to test the cyanophily of basidiospores. Basidiospores, basidia, pileipellis, stipitipellis and cystidia were illustrated by hand drawing.

In the description of basidiospores, the abbreviation [n/m/p] represents ‘n’ basidiospores measured from ‘m’ basidiomata of ‘p’ collections. Dimensions for basidiospores are given using notation of the form (a) b–c (d), while ‘a’ and ‘d’ mean the minimum and maximum, respectively, and ‘b–c’ contains a minimum of 90% of the measured values. The Q value represents the length/width ratio of a basidiospore inside view, and Qm value indicates average Q ± standard deviation. The terminology for denoting the shape of basidiospores followed Bas (1969).

The scanning electron microscopy (SEM) was also applied to observe the basidiospores ornamentation. Fragment of dry lamellae tissue samples were securely fastened to aluminum stubs and coated gold palladium before observed under a TESCAN CLARA Xplore 30 (Brno, Czech Republic) SEM.

### ﻿DNA extraction, PCR amplification and sequencing

Total genomic DNA was extracted using the Fungal DNA Mini Kit (Omega Bio-Tek, Norcross, USA) following the manufacturer’s instructions. For the PCR amplifications, the following primers were employed: (1) ITS5 and ITS4 (White et al. 1990) were used for the internal transcribed spacer (ITS); (2) LR0R and LR5 (Vilgalys and Hester 1990) for the nuclear ribosomal large subunit (LSU); (3) EF1-983F, EF1-1953R and EF1-1567R (Matheny et al. 2007), or the newly designed primers EF1-Laccaria-F1 (5’-ATGGACACCACCAAGGTAAGA-3’) and EF1-Laccaria-R1 (5’-ACGTTGCCACG.

ACGAATAT-3’), EF1-Laccaria-F2 (5’-TGCCTTTGTCCCTATTTCCG-3’) and EF1-Laccaria-R2 (5’-GGGTGGTTGAGGACGATGAC-3’) for the translation elongation factor 1-α (*TEF1*); (4) bRPB2-6F and bRPB2-7.1R (Matheny 2005), or the newly designed primers RPB2-Laccaria-F1 (5’-ACCATCACAAACGGTCTCA-3’) and RPB2-Laccaria-R1 (5’-CACCCTTTACCAGATGTTCC-3’), RPB2-Laccaria-F (5’-CTGAAGGTCAAGCCTGTGG-3’) and RPB2-Laccaria-R (5’-ACTTTGCTGTAGGC.

GAGAAT-3’) for polymerase II second largest subunit (*RPB2*). The new primers were designed using Primer 5.0.

The PCR mixtures were composed of 1 × PCR buffer, 1.5 mM MgCl_2_, 0.2 mM dNTPs, 0.4 μm forward primer, 0.4 μm reverse primer, 1.25U of *Taq* polymerase (CWBIO, Jiangsu, China), and 1 μL of DNA template in a total volume of 25 μL. Amplification reactions were performed with the following program: initial denaturation at 94 °C for 5 min, 35 cycles at 94 °C for 30 s, 52 °C (LSU and *TEF1*) or 54 °C (ITS and *RPB2*) for 30 s, and 72 °C for 30 s (ITS and LSU) or 45 s (*RPB2* and *TEF1*), and a final extension at 72 °C for 8 min (He et al. 2023). The products were subjected to electrophoresis on a 2% agarose gel, and the positively identified ones were sent to the Changsha branch of Youkang Biotechnology Co., Ltd. (Zhejiang, China) for sequencing.

### ﻿Sequence alignment and phylogenetic analysis

The sequences (Table 1) were aligned with the strategy FFT-NNS-I in the software MAFFT v7.511 (Katoh and Standley 2016). As shown in Table 1, a total of 94 sequences (28 ITS, 23 LSU, 19*TEF1* and 24*RPB2*) were newly generated in the present study. The intronic regions of *TEF1* and *RPB2* were manually excised. The ambiguously aligned regions of ITS and LSU were removed in Gblocks v0.91b (Castresana 2000).

A four-locus matrix for *Laccaria* (Suppl. material 1) was generated by SEQUENCEMATRIX 1.7.8 (Vaidya et al. 2011). The alignment contains 3423 positions from 215 samples, partitioned as follows: 1–631 (ITS), 632–1,510 (LSU), 1,511–2,402 (*TEF1*), and 2,403–3,423 (*RPB2*). The alignment has been deposited in TreeBASE (http://www.treebase.org/treebase/) under submission ID 32143. According to the AIC criterion in MRMODELTEST v2.4 (Nylander 2004), GTR+I+G was selected as the best-fit model for the four loci. Bayesian inference (BI) analysis was performed in MRBAYES v3.2.7 (Ronquist and Huelsenbeck 2003), with the GTR+I+G model for each partition, two simultaneous runs, four Markov Chain Monte Carlo (MCMC) chains, and sampling every 100 generations. After 20 million generations, the standard deviation of split frequencies was below 0.01. The first 25% generations were discarded as burn-in and the convergence was visually assessed by TRACER v1.7.2 (Rambaut et al. 2018). ML analysis with 1000 bootstrap replicates was computed in RAXML v8.0.20 (Stamatakis 2014), using the GTR+I+G model for each partition. Separate phylogenetic analyses based on the four loci were also conducted to assess potential conflicts, with the procedure described above.

## ﻿Results

### ﻿Phylogenetic analysis

**Figure 1.**
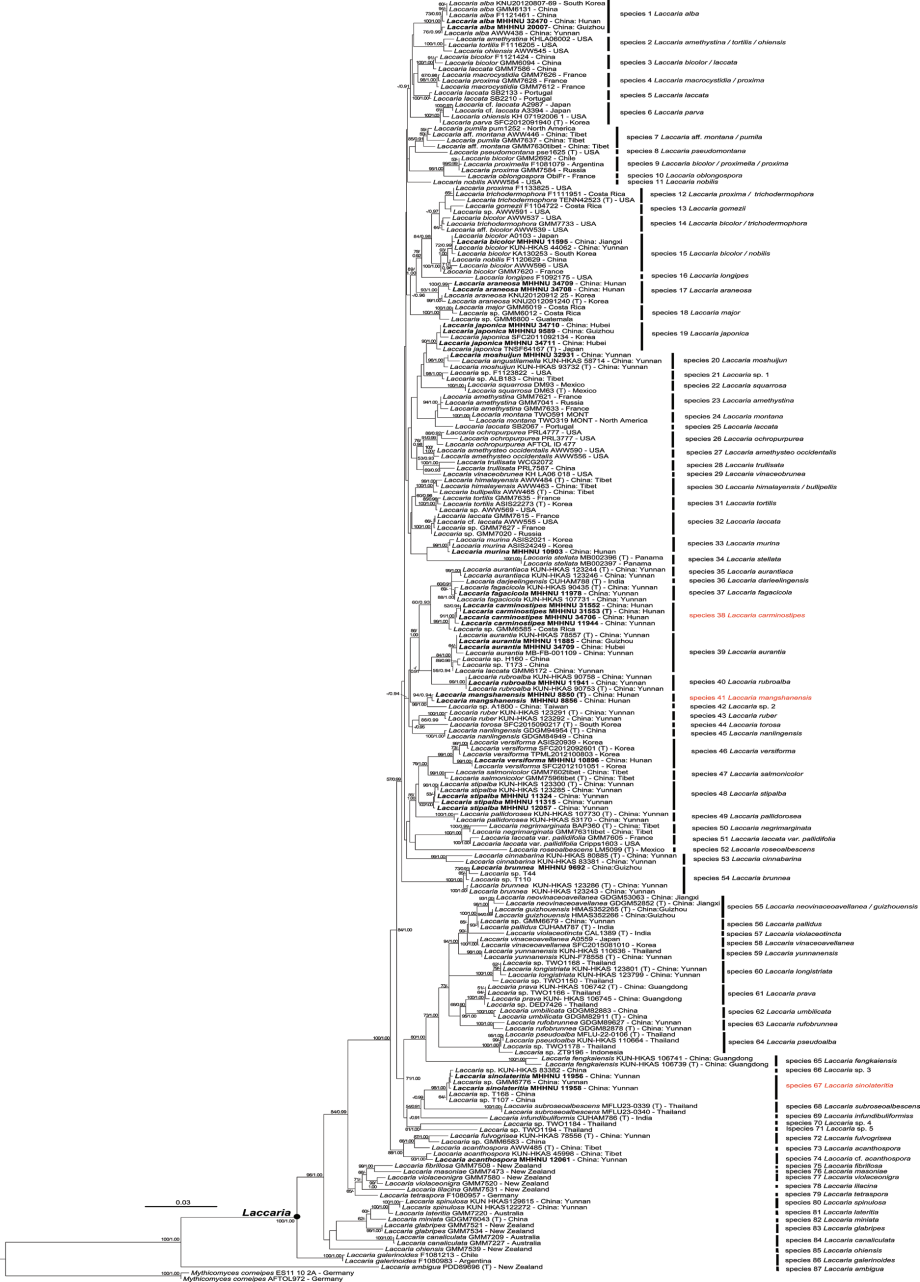
ML analysis of *Laccaria* based on ITS-LSU-*TEF1*-*RPB1* sequence data. Bootstrap values (BP) ≥ 50% from ML analysis and Bayesian posterior probabilities (PP) ≥ 0.90 from BI analysis are shown at nodes. Newly generated sequences are highlighted in bold. GenBank accession numbers of sequences and their geographic origins are shown. Taxa marked with (T) represent type specimens.

The phylogenetic trees of the genus *Laccaria* were constructed based on a four-locus matrix (ITS-LSU-*TEF1*-*RPB2*). As the topologies resulting from ML and BI analyses are consistent, only the ML tree is displayed (Fig. 1). Phylogenetic trees based on ITS, LSU, *TEF1* and *RPB2*, respectively, are provided in Suppl. material 2: figs S2–S5, which show no conflicts with the four-locus tree.

According to the phylogenetic tree, 87 species were recognized. The samples of the three newly discovered species described below, namely *L. carminostipes* (Species 38, 99% BP, 1.00 PP), *L. mangshanensis* (Species 41, 94% BP, 0.94 PP), and *L. sinolateritia* (Species 67, 98% BP, 1.00 PP) formed three independent clades. *Laccaria carminostipes* (MHHNU 11944,31552, 31553 and 34706) was recovered as sister (60% BP, 0.93 PP) to the clade (60% BP, 0.91 PP) containing *L. darjeelingensis* A. Thapa & K. Acharya, *L. fagacicola* Y.Y. Cui et al., and *L. aurantiaca* S.M. Tang et al. These species further formed a clade (60% BP, 0.93 PP), as sister (86% BP, 1.00 PP) to the clade (59% BP, 0.94PP) containing *L. aurantia* and *L. rubroalba* X. Luo et al. *Laccaria mangshanensis* (MHHNU 8850 and 8856) and the Chinese sample A1800 formed a clade with strong support (96% BP, 1.00 PP). *Laccaria sinolateritia* (MHHNU 11956 and MHHNU 11958) clustered together with *L. subroseoalbescens* S.M. Tang & S.H. Li with very low support (40% BP, 0.99 PP). Our Chinese samples MHHNU 9589, 34710, and 34711 formed a strongly supported clade with the holotype of *L. japonica* with minimal genetic distance (Species 19, 90% BP, 1.00 PP), which indicated that they should belong to the same species. Similarly, the Chinese sample MHHNU 10896 clustered with four Korean samples of *L. versiforma* (Species 46, 99% BP, 1.00 PP), which indicated that they could be conspecific.

The other samples analyzed in this work matched seven known species: *L. alba* (Species 1, 100% BP, 1.00 PP), *L. bicolor* (Maire) P.D. Orton (Species 15, 93% BP, 1.00 PP), *L. araneosa* H.J. Cho & Y.W. Lim (Species 17, 93% BP, 1.00 PP), *L. moshuijun* F. Popa & Zhu L. Yang (Species 20, 96% BP, 1.00 PP), *L. murina* S. Imai (Species 33, 99% BP, 1.00 PP), *L. fagacicola* (Species 37, 88% BP, 1.00 PP), *L. aurantia* (Species 39, 84% BP, 1.00 PP), *L. rubroalba* (Species 40, 99% BP, 1.00 PP), *L. stipalba* S.M. Tang et al. (Species 48, 100% BP, 1.00 PP), and *L. brunnea* S.M. Tang et al. (Species 54, 100% BP, 1.00 PP).

### ﻿Taxonomy

#### ﻿ Laccaria carminostipes

Taxon classificationFungiAgaricalesHydnangiaceae

Y.D. Xu & Z.M. He sp. nov.

C47C7829-3954-5964-ACF8-5E7AC8AC03FE

Fungal Names: FN 572506

Figs 2a–c, 3, 6a, b

##### Etymology.

‘*carminostipes*’ (Latin), referring to the carmine stipe surface.

**Figure 2.**
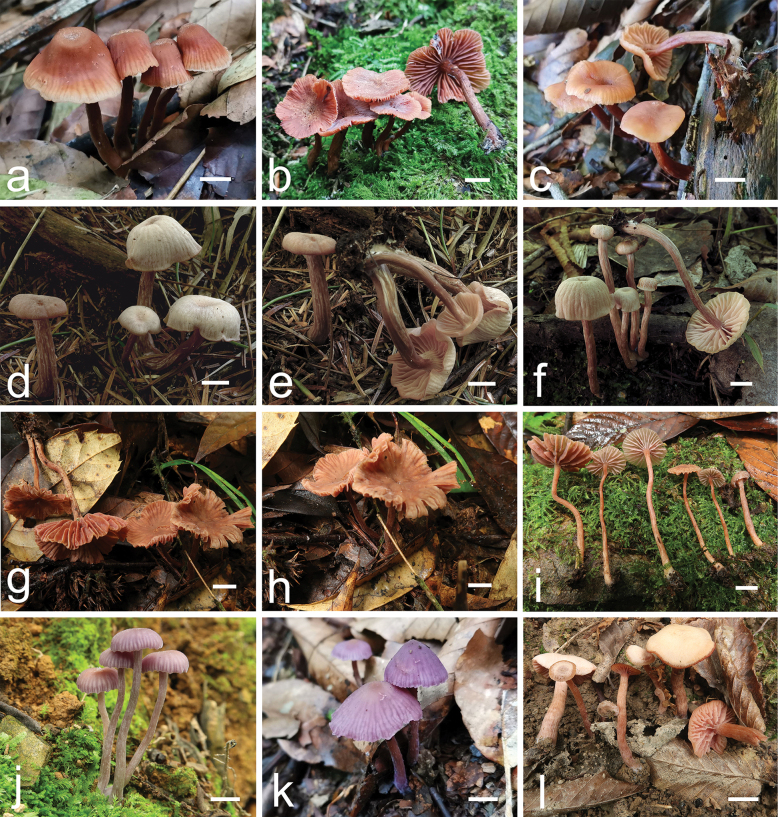
Basidiomata of *Laccaria carminostipes* (a. MHHNU 31553; b. MHHNU 31552; c. MHHNU 34706, respectively); *L. mangshanensis* (d, e. MHHNU 8850; f. MHHNU 8856, respectively); *L. sinolateritia* (g, h. MHHNU 11956; i. MHHNU 11958, respectively); *L. japonica* (j. MHHNU 9589; k. MHHNU 34711, respectively); *L. versiforma* (l. MHHNU 10896). Scale bars: 1 cm.

##### Diagnosis.

*L. carminostipes* exhibits a red-brown to orange-brown, translucent-striate pileus, salmon-pink adnate lamellae, a carmine equalstipe, globose to subglobose echinulate basidiospores, and a loosely interwoven pileipellis.

**Figure 3.**
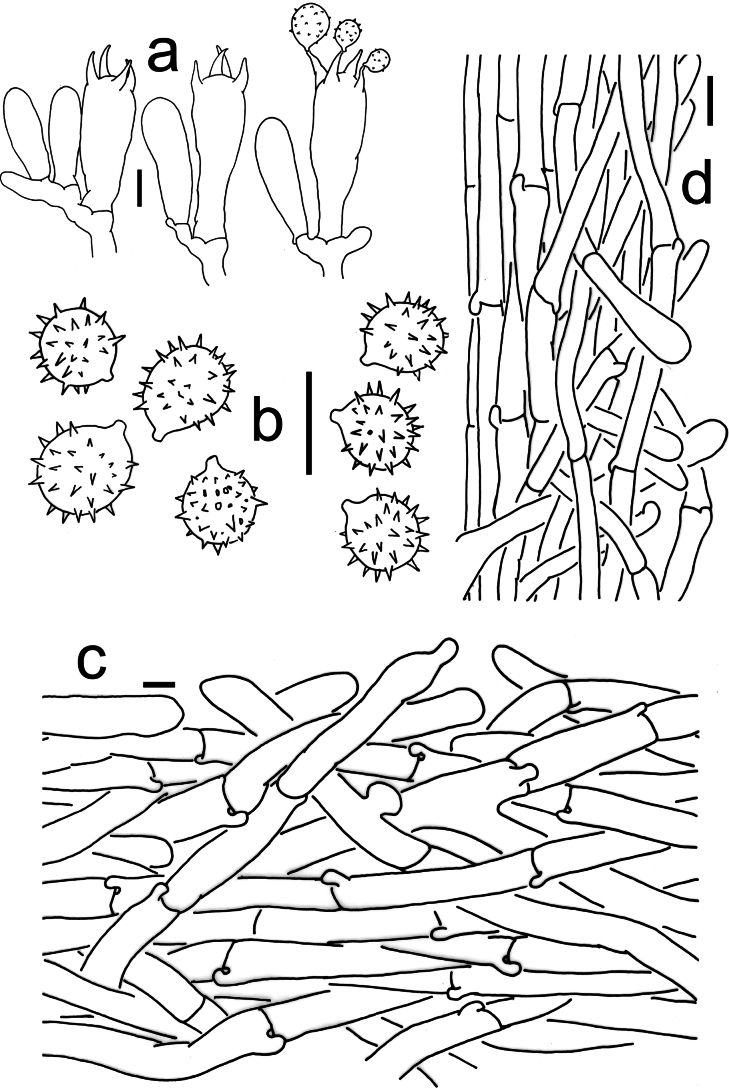
Microscopic features of *Laccaria carminostipes* (MHHNU 31553, holotype). a. Basidia; b. Basidiospores; c. Pileipellis; d. Stipitipellis. Scale bars: 10 μm.

##### Type.

China • Hunan Province: Sangzhi County, Badagongshan National Nature Reserve, at 29.65000°N, 109.695833°E, alt. 1742 m, in broad forest with trees of Fagaceae and bamboo, 29 July 2019, Z.H. Chen 31553 (MHHNU 31553, holotype).

##### Description.

Basidiomata small to medium-sized. Pileus 15–45 mm in diam, applanate to plano-concave, umbilicate, surface always fibrillose, sometimes missing, red-brown (7B6–7) to orange-brown (6A5–7), becoming darker with age, subhygrophanous; margin slightly translucent-striate. Lamellae adnate, distant, salmon-pink (7A3), narrow (about 2 mm high), entire, ventricose. Stipe 70–90 × 3–7 mm, central, equal, hollow, often slightly flexible, carmine (7C6–8); surface with indistinct to distinct whitish fibrillose coating; base with white (1A1) tomentum. Context thin, whitish (2A1) to brownish (5A2).

Basidiospores [100/4/4] (6.5) 7–9 (9.5) × (6) 6.5–8 (8.5) μm, Q = (0.88) 0.96–1.18 (1.33), Qm = 1.04 ± 0.08, mostly globose to subglobose, sometimes broadly ellipsoid, inamyloid, cyanophilous, echinulate; spines up to 1 μm long, ≤ 1 μm broad at base, distant; hilar appendix 1.0–1.8 μm long, prominent, truncate. Basidia 38–46 × 10–13 μm, clavate, mostly 4-spored, rarely 2-spored; sterigmata 6–8 μm long. Pleurocystidia and cheilocystidia not seen. Lamellar trama regular to subregular, composed of filamentous hyphae 3–6 μm wide. Pileipellis a cutis; hyphae loosely interwoven, thin-walled, cylindrical, 8–10 μm wide, in place with erect ends, with a brownish (3A2) intracellular pigment. Stipitipellis a cutis, composed of parallel to interwoven, thin-walled, cylindrical hyphae 3–8 μm wide, with some exserted inflated ends. Clamp connections present in all parts of basidiomata.

##### Ecology.

Always gregarious, under the trees of Fagaceae, in montane coniferous and broad-leaved mixed forests; summer (Jul.–Aug.).

##### Distribution.

Known from the subtropical zones of China.

##### Additional specimen examined.

China • Hunan Province, Sangzhi County, Badagongshan National Nature Reserve, at 29.65000°N, 109.695833°E, alt. 1740 m, 29 July 2019, Z.H. Chen 31552 (MHHNU 31552); • Sangzhi County, Badagongshan National Nature Reserve, at 29.755000°N, 109.762500°E, alt. 1592 m, under trees of Fagaceae,27 July 2020,Z.H. Chen 34706 (MHHNU 34706); • Yunnan Province, Jingdong Yi Autonomous County, Ailao Mountain, at 23.490278°N, 100.273333°E, alt. 2550 m, under trees of *Quercus*, 8 August 2024, P. Zhang 5444 (MHHNU 11944).

##### Notes.

*Laccaria rubroalba* and *L. carminnostipes* are characterized by medium-sized reddish basidiomata with a transluscent-striate pileus, but the former can be distinguished from the latter by having longer spines (1.2–2.7 μm long in *L. rubroalba* vs. up to 1 μm long in *L. carminnostipes*) and the presence of pleurocystidia and cheilocystidia (Luo et al. 2016). *Laccaria cinnabarina* J. Li & Y.Y. Cui is similar to *L. carminnostipes* by the reddish-brown stipes, but differs by its larger pileus (10–90 mm in *L. cinnabarina*vs. 15–45 mm in *L. carminnostipes*) and stronger spines (2 × 2 μm in *L. cinnabarina*vs. 1 × 1 μm in *L. carminnostipes*) (Li et al. 2024). *Laccaria macrobasidia* H.J. Cho & Y.W. Lim may sometimes be confused with *L. carminnostipes*, due to the similarity in size and color of their basidiomata. However, the two species can be differentiated based on the following diagnostic characteristics: basidiospores (9–11 × 8–10 μm in *L. macrobasidia* vs. 7–9 × 6.5–8 μm in *L. carminnostipes*), basidia (52–80 × 11–15 μm in *L. macrobasidia* vs. 38–46 × 10–13 μm in *L. carminnostipes*), pleurocystidia (present in *L. macrobasidia*, absent in *L. carminnostipes*), and the occurrence (*L. macrobasidia*in temperate forests, *L. carminnostipes* in subtropical forests) (Cho et al. 2020).

According to our phylogenetical analysis (Fig. 1), *L. carminostipes* (Species 38) could be most closely related to *L. fagacicola* (Species 37), *L. darjeelingensis* (Species 36), and *L. aurantiaca* (Species 35). *Laccaria fagacicola* can be distinguished from *L. carminostipes* by the presence of abundant cheilocystidia (Cui et al. 2021). *Laccaria darjeelingensis* differs by its dull red pileus and possesses both pleurocystidia and cheilocystidia (Thapa et al. 2024). *Laccaria aurantiaca* displays longer spines (0.7–1.8 μm vs. ≤ 1.0 μm in *L. carminostipes*) and produces pleurocystidia and cheilocystidia (Tang et al. 2025). Our phylogenetic analysis shows the placement of the sample GMM 6585 within the clade of *L. carminostipes* (Species 38, 99% BP, 1.00 PP, pairwise identity values of ITS = 99.84%), suggest that this specimen might be *L. carminostipes*.

#### ﻿ Laccaria mangshanensis

Taxon classificationFungiAgaricalesHydnangiaceae

Y.D. Xu & Z.M. He sp. nov.

A583755F-1E1D-5D26-B8FB-B2779C6CF47F

Fungal Names: FN 572507

Figs 2d–f, 4, 6c, d

##### Etymology.

‘*mangshanensis*’ referring to the locality of the holotype.

**Figure 4.**
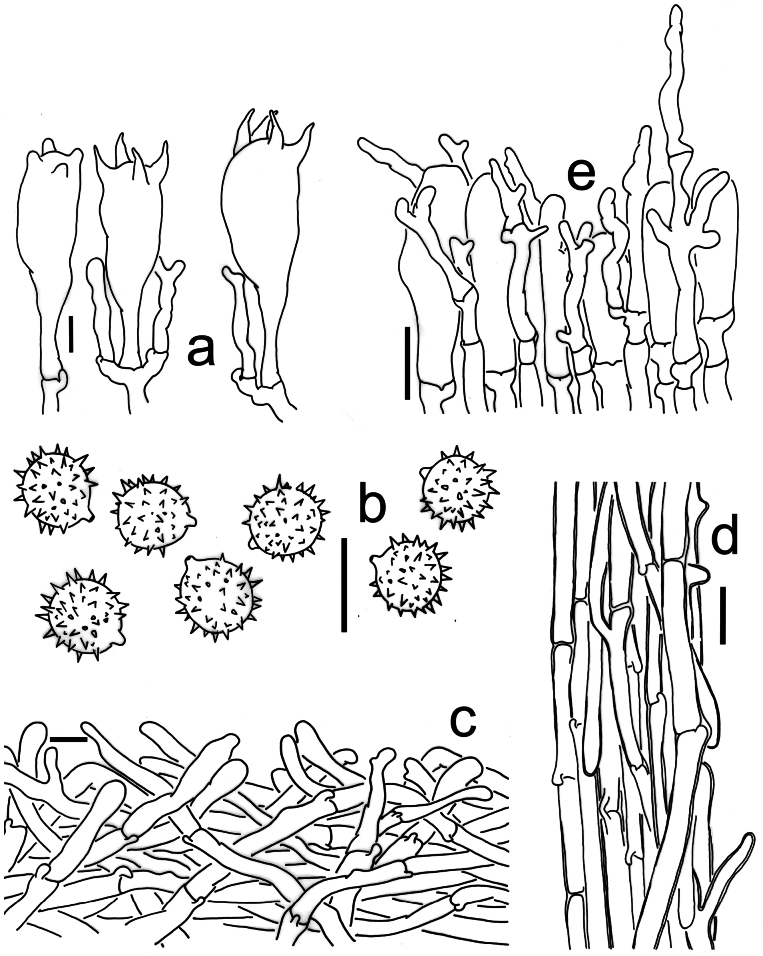
Microscopic features of *Laccaria mangshanensis* (MHHNU 8850, holotype). a. Basidia; b. Basidiospores; c. Pileipellis; d. Stipitipellis; e. Cheilocystidia. Scale bars: 10 μm.

##### Diagnosis.

*Laccaria mangshanensis* has a pale rosy, hemispherical, radially translucent-striate pileus, pinkish adnate lamellae, a reddish equal stipe, globose to subglobose echinulate basidiospores, and cheilocystidia.

##### Type.

China • Hunan Province: Yizhang County, Mangshan National Nature Reserve, at 24.866667°N,112.721944°E, alt. 1200 m, 28 July 2016, P. Zhang 2350 (MHHNU 8850, holotype).

##### Description.

Basidiomata small. Pileus 10–30 mm in diam, convex to hemispherical, centrally depressed, surface tomentose, dry, not hygrophanous, reddish-brown (7A5) to rosy (7A6) when young, then fading to pink-beige (7A2); margin strongly striate, rugulose-striate or rugulose-sulcate, involute to inflexed. Lamellae adnate, distant, pinkish (7A2) to white (1A1), narrow (about 2 mm high), entire, ventricose. Stipe 20–70 × 2–5 mm, central, equal, hollow, obviously flexible, reddish (7B5) to dull red (7C6), surface with distinct whitish fibrillose coating, base with white (1A1) tomentum. Context thin, whitish (2A1).

Basidiospores [100/4/2] (6.5) 7–8.5 (10) × (6) 6.5–8.5 (9) μm, Q = (0.86) 0.93–1.18 (1.31), Qm = 1.05 ± 0.08, mostly globose to subglobose, thin-walled, inamyloid, cyanophilous, hyaline, echinulate, spines (0.5) 1–1.2 (1.5) μm long, 0.3–0.8 (1) in width, subdistant; hilar appendix 0.8–1.5 μm long, subtruncate. Basidia 38–47 × 10–13 μm, 4-spored, clavate, sharply narrowed, thin-walled, hyaline; sterigmata up to 9 μm long. Pleurocystidia lacking. Cheilocystidia 25–55 × 3–5 μm, filamentous to narrowly clavate, flexuose, thin-walled, hyphae, abundant. Lamellar trama regular to subregular; hyphae cylindrical, hyaline, thin- walled, 3–8 μm wide. Pileipellis a cutis, composed of thin-walled, interwoven, cylindrical hyphae 4–10 μm wide, with exserted ends, hyphae. Stipitipellis a cutis, composed of appressed, parallel, thin- to slightly thick-walled (ca. 0.5 μm) hyphae. Clamp connections present in all parts of basidiomata.

##### Ecology.

Single, in clusters or in groups, on soil, under the trees of Fagaceae, in subtropical montane coniferous and broadleaved mixed forest, summer (Jul.).

##### Distribution.

Known from Central China.

##### Additional specimen examined.

China • Hunan Province: Yizhang County, Mangshan National Nature Reserve, at 24.869722°N, 112.721944°E, alt. 1200 m, 29 July 2016, P. Zhang 2356 (MHHNU 8856).

##### Notes.

*Laccaria fengkaiensis* F. Liand *L. mangshanensis* share pale red to pastel red basidiomata, but the former can be distinguished from the latter by the larger pileus (50–90 mm pileus width vs. 10–30 mm in *L. mangshanensis*), smaller basidiospores (5–6 × 5–6 μm vs. 7–8.5 × 6.5–8.5 μm in *L. mangshanensis*), and the presence of pileocystidia (Li 2020). However, the phylogenetic analysis (Fig. 1) suggests that *L. mangshanensis* represents a well-supported clade with strong support (Species 41, 94% BP, 0.94 PP), and is clearly separated from *L. fengkaiensis* (Species 65).

#### ﻿ Laccaria sinolateritia

Taxon classificationFungiAgaricalesHydnangiaceae

Y.D. Xu & Z.M. He sp. nov.

7DE42960-73A2-5B48-8C29-C5CB0A941462

Fungal Names: FN 572508

Figs 2g–i, 5, 6e, f

##### Etymology.

‘*sino*’ referring to China, ‘*lateritia*’ (Latin) referring to its red to brownish orange fruiting body.

**Figure 5.**
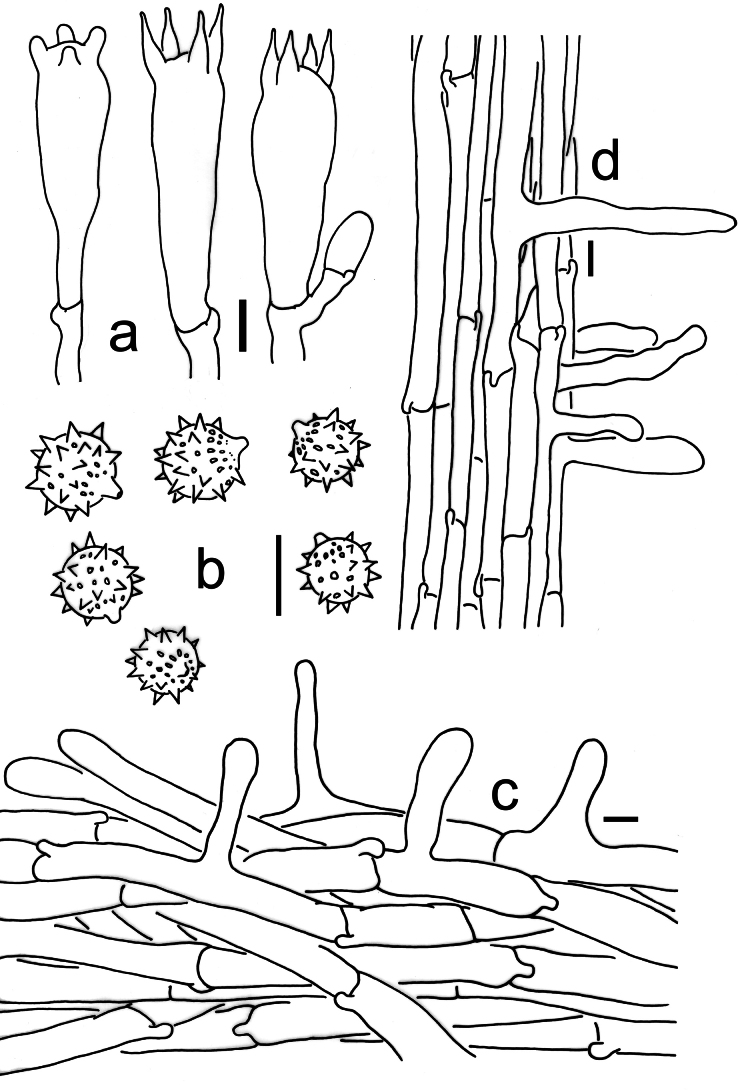
Microscopic features of *Laccaria sinolateritia* (MHHNU 11956, holotype). a. Basidia; b. Basidiospores; c. Pileipellis; d. Stipitipellis. Scale bars: 10 μm.

**Figure 6.**
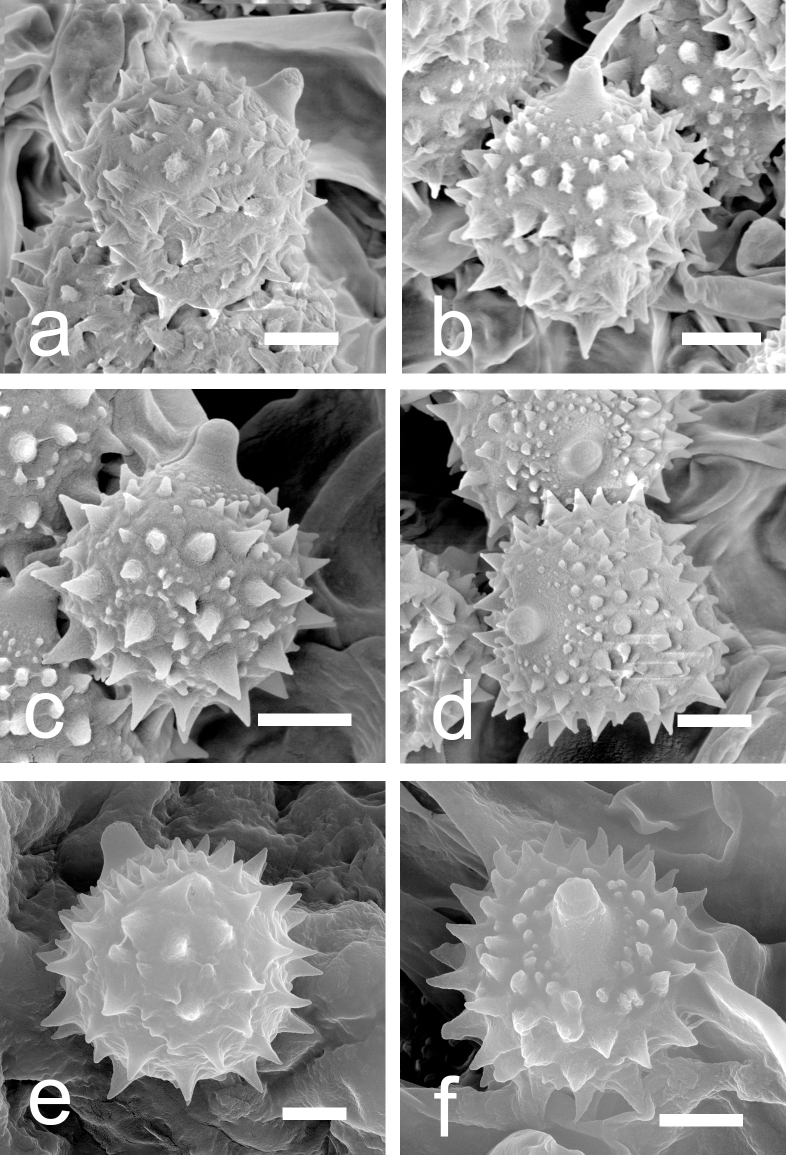
Basidiospores of described species under SEM. a, b. *Laccaria carminostipes* (MHHNU 31553, holotype); c, d. *L. mangshanensis* (MHHNU 8850, holotype); e, f. *L. sinolateritia* (MHHNU 11956, holotype). Scale bars: 2 μm.

##### Diagnosis.

*Laccaria sinolateritia*is characterized by brownish red basidiomata, globose to subglobose echinulate basidiospores, and the presence of pileocystidia and caulocystidia.

##### Type.

China • Yunnan Province: Jingdong Yi Autonomous County, Ailaoshan, at 24.904444°N,101.059444°E, alt. 2500 m, 8 August 2024, P. Zhang 5456 (MHHNU 11956, holotype).

##### Description.

Basidiomata small to medium-sized. Pileus 10–40 mm in diam, at first convex, then plano-concave to concave; surface tomentose, brownish red (6B4), subhygrophanous; translucent-striate or rugulose-sulcate, straight, undulate with age. Lamellae distant, entire, adnate, broad (about 5 mm high), ventricose, concolorous with pileus surface. Stipe 35–50 × 1–3 mm, brownish red (6B4), equal, broadly fistulose; surface covered with whitish (2A1) longitudinal fibrils; base with a whitish (2A1) mycelium. Context brownish (6A4).

Basidiospores [100/4/2] (7) 7.5–10 (10.5) × (7) 7.5–10 (10.5) μm, Q = (0.90) 0.91–1.07 (1.11), Qm = 1.00 ± 0.05, mostly globose, hyaline, echinulate; spines 2–2.5 (3) μm long, ca. (1) 1.5–2 μm wide at base, crowded; hilar appendix 1.3–2.5 long, prominent, subtruncate. Basidia 40–58 × 12–14 μm, with 4 sterigmata up to 12 μm long, hyaline, subcylindrical to clavate. Pleurocystidia and cheilocystidia not seen. Lamellar trama subregular to interwoven; hyphae cylindrical, hyaline, thin-walled, 2–6 (7) μm wide. Pileipellis a cutis, hyphae cylindrical, 8–10 (12) μm in diameter with some hyphal ends, subregular to interwoven. Pileocystidia abundant, 25–45 × 5–15 μm, cylindrical to clavate, vertically to subvertically arrange, scattered to aggregating into clusters on the pileus surface. Stipitipellisa cutis, composed of cylindrical hyphae 5–10 (11.5) μm wide with abundant caulocystidia, thin- to thick-walled (≤0.5 μm). Caulocystidia 37.5–55 (67.5) × 7.5–9.5 (10) μm, clavate, scattered to aggregating into clusters on the stipitipellis surface. Clamp connections present in all parts of basidiomata.

##### Ecology.

Gregarious, under the trees of Fagaceae, in subtropical montane forests, summer (Aug.).

##### Distribution.

Known from the subtropical zones of China.

##### Additional specimen examined.

China • Yunnan Province: Jingdong Yi Autonomous County, Ailaoshan, at 24.904444°N, 101.059444°E, alt. 2500 m, 8 August 2024, P. Zhang 5458 (MHHNU 11958).

##### Notes.

Macroscopically, *L. lateritia* Malençon and *L. sinolateritia* are easily confused. For instance, they share similar-sized basidiomata, reddish-brown pileus and similar-sized basidiospores (7.5–10.5 × 7.5–10.5 µm in *L. lateritia* vs. 7.5–10 × 7.5–10 µm in *L. sinolateritia*). However, *L. lateritia* can be distinguished by the production of 2-spored basidia, shorter spines (±1 μm high vs. 2–2.5 μm high in *L. sinolateritia*) and the presence of cheilocystidia (Cooper 2015). Additionally, *L. darjeelingensis* resembles *L. sinolateritia* in its red basidiomata, but differs in having smaller basidiospores (5.9–7.6 × 5.9–7.6 µm vs. 7.5–10 × 7.5–10 µm in *L. sinolateritia*) and abundant flexuous pleurocystidia and cheilocystidia (Thapa et al. 2024).

According to the phylogenetical analysis (Fig. 1), *L. subroseoalbescens* (species 68) and *L. infundibuliformis* (species 69) are related to *L. sinolateritia* (species 67), and form a clade with low support (54%BP, 0.91PP). *Laccaria subroseoalbescens*is characterized by a yellow pileus, the presence of cheilocystidia and pleurocystidia, and the absence of pileocystidia (Tang et al. 2024). *Laccaria infundibuliformis* differs in its smaller pileus (8–27 mm in diam vs. 10–40 mm in diam in *L. sinolateritia*), smaller basidia (29–50 × 6–9 µm vs. 40–58 × 12–14 μm in *L. sinolateritia*), and the absence of pileocystidia (Thapa et al. 2024).The four-locus phylogenetical analysis also supports the identification of the four Chinese samplesKUN-HKAS83382, GMM6776, T168, and T107 as *L. sinolateritia* (pairwise identity values of ITS = 99.39%–100%).

## ﻿Discussion

Our study presents three new species of *Laccaria*, viz. *L. carminostipes*, *L. mangshanensis* and *L. sinolateritia* based on a comprehensive analysis of morphological and molecular phylogenetic data (Aime et al. 2021). The four-locus phylogenetic tree strongly supports that they are monophyletic clades separated from other *Laccaria* species (Fig. 1). In addition, *L. japonica* and *L. versiforma* is formally reported here for the first time in China.

*Laccaria carminostipes*, *L. mangshanensis*, and *L. sinolateritia* were collected from Yunnan and Hunan Provinces, regions known for their rich ecological resources and high fungal diversity. *Laccaria carminostipes*is can be distinguished from other *Laccaria* species by its distinctive carmine-colored stipe. *L. mangshanensis* is characterized by a rosy pileus and the presence of abundant cheilocystidia. And *L. sinolateritia* is characterized by its brownish-red basidiomata and the presence of abundant pileocystidia and caulocystidia.

Regarding *L. japonica* (Vincenot et al. 2017), the Chinese specimens, MHHNU 9589, 34710 and 34711 (Fig. 2j, k), resemble to this species, exhibiting small to medium-sized, bright purple basidiomata, adnate lamellae, and similar basidiospores size (8–10 × 8–10 µm in our specimens, 9–10 × 7–9 µm in *L. japonica*). Our phylogenetic analysis (Fig. 1) further indicates that these samples form a well-supported clade together with the holotype of *L. japonica* (90% BP, 1.00 PP, pairwise identity values of ITS = 99.68%–99.83%). Similarly, the Chinese specimen MHHNU 10896 (Fig. 2l) has small to medium-sized basidiomata, featuring pale brown to pinkish brown pileus, brown stipes, pinkish lamellae, and echinulate basidiospores measuring 7–10 × 7–9.5 µm. These features are entirely consistent with the description of *L. versiforma* provided by Cho et al. (2018). Furthermore, in the four-locus phylogenetic tree (Fig. 1), the specimen MHHNU 10896 clusters together with four Korean specimens of *L. versiforma* with strong support (99% BP, 1.00 PP). The ITS sequence of MHHNU 10896 exhibits a similarity of 98.12%–99.64%. Both *L. japonica* and *L. versiforma* occur in temperate or subtropical forest. *Laccaria japonica*was previously documented in temperate forests of *Salix reinii* Franch. & Sav.in Japan (Vincenot et al. 2017), while our Chinese collections of *L. japonica* were found in subtropical forests of Fagaceae. In Korea, *L. versiforma* was found in temperate forests of *Quercus* and *Pinus densiflora* Siebold &Zucc. (Cho et al. 2018), while our specimen of *L. versiforma* was found in subtropical forests of *Quercus*.

The phylogram recovers 87 species of *Laccaria*, yet the infrageneric classifications for some species within this genus remains indeterminate. Our specimen MHHNU 12061 could be *L. acanthospora*, based on its orange pileus, and basidiospores measuring 8–10 × 7–10 µm (7–10 × 7–10 µm in *L. acanthospora*, Wilson et al. 2013). However, the sample MHHNU 12061 exhibits a relatively long genetic distance from the holotype of *L. acanthospora* (AWW485, Fig. 1) and possesses a larger pileus measuring 20–30 mm in width (Suppl. material 2: fig. S1l, 4–15 mm in Wilson et al. 2013). Because we have only one specimen, it remains uncertain whether these morphological differences reflect intraspecific variation or are significant enough to warrant the recognition of a distant species.

*Laccaria bicolor* was originally described from Europe (Maire 1937). According to our phylogenetic analysis (Fig. 1), the sequences named *L. bicolor* were clustered into four clades (Species 3, 9, 14 and 15). Due to the lack of the sequence of the type specimen for *L. bicolor*, the exact phylogenetic position of this species remains uncertain. Our Chinese specimen MHHNU 11595 (Suppl. material 2: fig. S1e) clusters together with a French specimen of *L. bicolor* with full support in our phylogenetic analysis (Fig. 1). Since their ITS pairwise identity value is 97.91%, the Chinese specimen could be a cryptic species. *Laccaria guizhouensis* (Zhang et al. 2024) and *L. neovinaceoavellanea* (Zhang et al. 2023) are likely conspecific, as their holotypes (HMAS 352265 and GDGM52852) form a highly supported clade (98% BP, 1.00 PP) with an ITS pairwise identity value of 99.44%. Similarly, *L. himalayensis* and *L. bullipellis* may also be conspecific, given their high ITS pairwise identity values of 99.17% and stable clustering with a full support.

Based on the four-locus phylogenetic and morphological analyses (Fig. 1, Suppl. material 2: fig. S1), we also identified nine other previously described species: *L. alba*, *L. araneosa*, *L. aurantia*, *L. brunnea*, *L. fagacicola*, *L. moshuijun*, *L. murina*, *L. rubroalba*, and *L. stipalba*. Both *L. alba* and *L. aurantina*, were first reported in Yunnan Province. In this study, *L. alba* was also found in Hunan and Guizhou Provinces, and *L. aurantina* was discovered in Hubei and Guizhou Provinces. *Laccaria araneosa*, originally described in Korean temperate forests (Cho et al. 2018), and later also reported in Jilin Province, temperate China (Wang et al. 2022). We also found this species in Hunan Province, subtropical China.

*Laccaria brunnea* was first recorded in Yunnan Province, China. This species is characterized by its brownish basidioma with salmon lamellae, and the presence of pleurocystidia and cheilocystidia (Tang et al. 2025). Our specimen MHHNU 9692 (Suppl. material 2: fig. S1d), collected from Guizhou Province, China, also has a gray to brownish pileus, salmon lamellae, and globose to subglobose, echinulate basidiospores, showing morphological resemblance to *L. brunnea*. In our four-locus phylogenetic tree (Fig. 1), the samples MHHNU 9692, T44, and T110 cluster together with the holotype (KUN-HKAS 123286) of *L. brunnea* (100% BP, 1.00 PP, pairwise identity values of ITS = 98.37%–98.91%), confirming the identification of sample MHHNU 9692 as *L. brunnea*. Notably, both pleurocystidia and cheilocystidia were found to be absent in our specimen MHHNU 9692.*Laccaria stipalba*is characterized by its dark orange to grayish pink pileus, distinctive white stipe, and globose echinulate basidiospores, and was originally described in Yunnan Province (Tang et al. 2025). During 2022 to 2024, we also collected three specimens MHHNU 11314, 11324 and 12057 from Yunnan Province, China, which exhibit the same microfeatures (Suppl. material 2: fig. S1j, k and l). In our phylogenetic analysis (Fig. 1), these three samples cluster with the holotype of *L. stipalba* (KUN-HKAS 123285) with full support (pairwise identity values of ITS = 99.65%–99.83%), confirming that these samples should be identified as *L. stipalba*. It should be pointed out that the basidiospores size of our specimens is slightly larger (7–10 × 7–10 μm vs. 5.8–8.4 × 5.5–8.1 μm in Tang et al. 2025), and pleurocystidia and cheilocystidia were not found in our examination.

## Supplementary Material

XML Treatment for Laccaria carminostipes

XML Treatment for Laccaria mangshanensis

XML Treatment for Laccaria sinolateritia

## ﻿Additional information

### Use of AI

No use of AI was reported.

### Author ORCIDs

You-Di Xu https://orcid.org/0009-0004-8112-639X

Ping Zhang https://orcid.org/0000-0002-8751-704X

Zuo-Hong Chen https://orcid.org/0000-0001-7359-0257

Zheng-Mi He https://orcid.org/0000-0001-8754-3427

### Data availability

The DNA sequences obtained in this study have been submitted to GenBank.

## Supplementary materials

Supplementary material 1 Dataset of four-locus Data type fas https://binary.pensoft.net/file/1436806This dataset is made available under the Open Database License (http://opendatacommons.org/licenses/odbl/1.0/). The Open Database License (ODbL) is a license agreement intended to allow users to freely share, modify, and use this Dataset while maintaining this same freedom for others, provided that the original source and author(s) are credited.You-Di Xu, Ping Zhang, Zuo-Hong Chen, Zheng-Mi He

Supplementary material 2 Supplementary images Data type pdf https://binary.pensoft.net/file/1436807This dataset is made available under the Open Database License (http://opendatacommons.org/licenses/odbl/1.0/). The Open Database License (ODbL) is a license agreement intended to allow users to freely share, modify, and use this Dataset while maintaining this same freedom for others, provided that the original source and author(s) are credited.You-Di Xu, Ping Zhang, Zuo-Hong Chen, Zheng-Mi He

## References

[B1] Aime MC Miller AN Aoki T Bensch K Cai L Crous PW Hawksworth DL Hyde KD Kirk PM Lücking R May TW Malosso E Redhead SA Rossman AY Stadler M Thines M Yurkov AM Zhang N Schoch CL (2021) How to publish a new fungal species, or name, version 3.0. IMA Fungus 12: 11. 10.1186/s43008-021-00063-1 PMC8091500 33934723

[B2] Berkeley MJ Broome CE (1883) Notices of British fungi> (1989–2027). Annals & Magazine of Natural History 12: 370–374. 10.1080/00222938309459647

[B3] Besson M Kühner R (1971) Ultrastructure de la paroisporique des *Laccaria* Berk. et Br. (Agaricales). Comptes Rendus Hebdomadaires des Séances de l’Académie des Sciences 272: 1078–1081.

[B4] Campi M Mancuello C Maubet Y Niveiro N (2017) *Laccaria fraterna* (Cooke & Mass.: Sacc.) Pegler, 1965 (Agaricales, Basidiomycota) associated with exotic Eucalyptus sp. in northern Argentina and Paraguay. Check List 13: 87–90. 10.15560/13.4.87

[B5] Castresana J (2000) Selection of conserved blocks from multiple alignments for their use in phylogenetic analysis. Molecular Biology and Evolution 17: 540–552. 10.1093/oxfordjournals.molbev.a026334 10742046

[B6] Cho HJ Park MS Lee H Oh SY Wilson AW Mueller GM Lim YW (2018) A systematic revision of the ectomycorrhizal genus *Laccaria* from Korea. Mycologia 110: 948–961. 10.1080/00275514.2018.1507542 30240340

[B7] Cho HJ Lee H Park MS Park KH Park JH Cho Y Kim C Lim YW (2020) Two new species of *Laccaria* (Agaricales, Basidiomycota) from Korea. Mycobiology 48: 288–295. 10.1080/12298093.2020.1786961 32952411 PMC7476507

[B8] Cooper J (2015) Mycological Notes – 30: A Preliminary Key to New Zealand species of *Laccaria*. Manaaki Whenua – Landcare Research. [Unpublished technical report]

[B9] Corrales A Wilson AW Mueller G Ovrebo M (2020) Novel *Laccaria* species from Juglandaceae forest in Panama with notes on their ecology. Frontiers in Microbiology 11: 1597. 10.3389/fmicb.2020.01597 PMC7380087 32765456

[B10] Cui YY Cai Q Li J Yang ZL (2021) Two new *Laccaria* species from China based on molecular and morphological evidence. Mycological Progress 20: 567–576. 10.1007/s11557-021-01698-5

[B11] Deepna Latha KD Raj KA Manimohan P (2019) *Laccaria violaceotincta*: A new species from tropical India based on morphology and molecular phylogeny. Phytotaxa 392: 140–146. 10.11646/phytotaxa.00.0.0

[B12] Dovana F Moreno G Para R Lavorato C Mucciarelli M (2021) Phylogenetic reappraisal and epitypification of *Laccaria macrocystidiata* (Hydnangiaceae, Basidiomycota). Phytotaxa 514: 129–139. 10.11646/phytotaxa.514.2.4

[B13] Guzmán G (2016) Los hongos de la Península de Yucatán (México) V. Nuevasobservaciones y nuevosregistros. Scientia Fungorum 3: 7–13. 10.33885/sf.2004.3.908

[B14] He ZM Chen ZH Bau T Wang GS Yang ZL (2023) Systematic arrangement within the family Clitocybaceae (Tricholomatineae, Agaricales): Phylogenetic and phylogenomic evidence, morphological data and muscarine-producing innovation. Fungal Diversity 123: 1–47. 10.1007/s13225-023-00527-2

[B15] Katoh K Standley DM (2016) A simple method to control over-alignment in the MAFFT multiple sequence alignment program. Bioinformatics 32: 1933–1942. 10.1093/bioinformatics/btw108 27153688 PMC4920119

[B16] Kornerup A Wanscher JH (1978) Methuen handbook of colour (3^rd^ edn.). Eyre Methuen, London, 252 pp.

[B17] Li F (2020) Two new species of *Laccaria* from South China, with a note on *Hodophilus glaberipes*. Mycological Progress 19: 525–539. 10.1007/s11557-020-01573-9

[B18] Li Y Li TH Yang ZL Bau T Dai YC (2015) Atlas of Chinese Macrofungal Resources. Central China Farmer’s Publishing House, Zhengzhou, 1400 pp.

[B19] Li J Che NJ Cui YY (2024) Three new species of *Laccaria* (Agaricales, Basidiomycota) from Southwest China (Yunnan) based on morphological and multi-gene sequence data. Frontiers in Microbiology 15: 1411488. 10.3389/fmicb.2024.1411488 PMC11335674 39171265

[B20] Luo X Ye L Chen J Karunarathna SC Xu JC Hyde KD Mortimer PE (2016) *Laccaria rubroalba* sp. nov. (Hydnangiaceae, Agaricales) from southwestern China. Phytotaxa 284: 041–050. 10.11646/phytotaxa.284.1.4

[B21] Maire R (1937) Fungi Catalaunici: Series altera. Publications de l’Institut de Botànica de Barcelona 3: 1–128.

[B22] Mao XL (2000) The Macrofungi in China. Henan Science and Technology Press, Zhengzhou, 718 pp.

[B23] Matheny PB (2005) Improving phylogenetic inference of mushrooms with *RPB1* and *RPB2* nucleotide sequences (*Inocybe*; Agaricales). Molecular Phylogenetics and Evolution 35: 1–20. 10.1016/j.ympev.2004.11.014 15737578

[B24] Matheny PB Wang Z Binder M Curtis JM Lim YW Nilsson H Hughes KW Hofstetter V Ammirati JF Schoch CL Langer E Langer G McLaughlin DJ Wilson AW Frøslev T Ge ZW Kerrigan RW Slot JC Yang ZL Baroni TJ Fischer M Hosaka K Matsuura K Seidl MT Vauras J Hibbett DS (2007) Contributions of *rpb2* and *tef1* to the phylogeny of mushrooms and allies (Basidiomycota, Fungi). Molecular Phylogenetics and Evolution 43: 430–451. 10.1016/j.ympev.2006.08.024 17081773

[B25] Mueller GM (1984) New North American species of *Laccaria* (Agaricales). Mycotaxon 20: 101–116. 10.5962/p.418792

[B26] Mueller GM (1991a) *Laccaria longipes*, a new North America species of the *Laccaria laccata* complex. Mycotaxon 40: 145–150.

[B27] Mueller GM (1991b) *Laccaria laccata* complex in North America and Sweden: Intercollection pairing and morphometric analyses. Mycologia 83: 578–594. 10.2307/3760213

[B28] Mueller GM Sundberg WJ (1981) A floristic study of *Laccaria* (Agaricales) in southern Illinois. Nova Hedwigia 34: 577–597.

[B29] Mueller GM Vellinga EC (1986) Taxonomic and nomenclatural notes on *Laccaria*, B. & Br. *Laccaria amethystea*, *L. fraterna*, *L. laccata*, *L. pumila*, and their synonyms. Persoonia Molecular Phylogeny and Evolution of Fungi 13: 27–43.

[B30] Nylander JAA (2004) MrModeltest version 2. Program distributed by the author. Evolutionary Biology Centre, Uppsala University. https://github.com/nylander

[B31] Osmundson TW Cripps CL Mueller GM (2005) Morphological and molecular systematics of Rocky Mountain alpine *Laccaria*. Mycologia 97: 949–972. 10.1080/15572536.2006.11832746 16596948

[B32] Popa F Rexer KH Donges K Yang ZL Kost G (2014) Three new *Laccaria* species from Southwest China (Yunnan). Mycological Progress 13: 1105–1117. 10.1007/s11557-014-0998-7

[B33] Popa F Jimenéz SYC Weisenborn J Donges K Rexer KH Piepenbring M (2016) A new *Laccaria* species from cloud forest of Fortuna, Panama. Mycological Progress 15: 19. 10.1007/s11557-015-1139-7

[B34] Rambaut A Drummond AJ Xie D Baele G Suchard MA (2018) Posterior summarisation in Bayesian phylogenetics using Tracer 1.7. Systematic Biology 67: 901–904. 10.1093/sysbio/syy032 29718447 PMC6101584

[B35] Ramos A Bandala VM Montoya L (2017) A new species and a new record of *Laccaria* (Fungi, Basidiomycota) found in a relict forest of the endangered Fagus grandifolia var. mexicana. MycoKeys 27: 77–94. 10.3897/mycokeys.27.21326 PMC5804296 29559819

[B36] Ronquist F Huelsenbeck JP (2003) MrBayes 3: Bayesian phylogenetic inference under mixed models. Bioinformatics 19: 1572–1574. 10.1093/bioinformatics/btg180 12912839

[B37] Simard SW (2009) The foundational role of mycorrhizal networks in self-organization of interior Douglas-fir forests. Forest Ecology and Management 258(Suppl.): S95–S107. 10.1016/j.foreco.2009.05.001

[B38] Singer R (1967) Notes sur le genre *Laccaria*. Bulletin Trimestriel de la Société Mycologique de France 83: 104–123.

[B39] Stamatakis A (2014) RAxML version 8: A tool for phylogenetic analysis and post-analysis of large phylogenies. Bioinformatics 30: 1312–1313. 10.1093/bioinformatics/btu033 24451623 PMC3998144

[B40] Tang SM Vadthanarat S Raghoonundon B Luo ZL Zhu XY Yu FM He J Li SH (2024) New species and new records of *Laccaria* (Agaricales, Basidiomycota) from Northern Thailand. MycoKeys 107: 189–217. 10.3897/mycokeys.107.127907 39169989 PMC11336393

[B41] Tang SM Zhao G Niu KY Li RY Yu FM Karunarathna SC Li L Hyde KD Su XJ Luo ZL (2025) Species diversity of edible mushrooms I—four new *Laccaria* species from Yunnan Province. Journal of Fungi 11: 189. 10.3390/jof11030189 PMC11942694 40137227

[B42] Thapa A Tamang J Acharya K (2024) Three new species of *Laccaria* (Hydnangiaceae) from India (Darjeeling Hills) based on molecular and morphological evidence. Current Microbiology 81: 79. 10.1007/s00284-023-03598-1 38281219

[B43] Vaidya G Lohman DJ Meier R (2011) Sequencematrix: Concatenation software for the fast assembly of multi-gene datasets with character set and codon information. Cladistics: The International Journal of the Willi Hennig Society 27: 171–180. 10.1111/j.1096-0031.2010.00329.x 34875773

[B44] van der Heijden MGA Martin FM Selosse M-A Sanders IR (2015) Mycorrhizal ecology and evolution: The past, the present, and the future. The New Phytologist 205: 1406–1423. 10.1111/nph.13288 25639293

[B45] Vilgalys R Hester M (1990) Rapid genetic identification and mapping of enzymatically amplified ribosomal DNA from several Cryptococcus species. Journal of Bacteriology 172: 4238–4246. 10.1128/jb.172.8.4238-4246 2376561 PMC213247

[B46] Vincenot L Popa F Laso F Donges K Rexer KH Kost G Yang ZL Nara K Selosse MA (2017) Out of Asia: Biogeography of fungal populations reveals Asian origin of diversification of the *Laccaria amethystina* complex, and two new species of violet *Laccaria*. Fungal Biology 121: 939–955. 10.1016/j.funbio.2017.08.001 29029701

[B47] Vizzini A Alvarado P Consiglio G Marchetti M Xu J (2024) Family matters inside the order Agaricales: Systematic reorganization and classification of incertae sedis clitocyboid, pleurotoid and tricholomatoid taxa based on an updated 6-gene phylogeny. Studies in Mycology 107: 67–148. 10.3114/sim.2024.107.02 38600959 PMC11003440

[B48] Wang L Yang ZL Liu JH (2004) Two new species of*Laccaria* (Basidiomycetes) from China. Nova Hedwigia 79: 511–517. 10.1127/0029-5035/2004/0079-0511

[B49] Wang K Liu DM Li GJ Liu TZ Xie ML Du Z Wei TZ (2022) Taxonomy of *Laccaria* in China based on the specimens collected in the HMAS. Journal of Fungal Research 20: 271–284. 10.13348/j.jfr.2022.1566

[B50] White TJ Bruns T Lee S Taylor J (1990) Amplification and direct sequencing of fungal ribosomal RNA genes for phylogenetics. In: Innis MA Gelfand DH Sninsky JJ White TJ (Eds) PCR protocols: a guide to methods and applications.Academic Press, San Diego, 315–322. 10.1016/b978-0-12-372180-8.50042-1

[B51] Wilson AW Hosaka K Perry BA Mueller GM (2013) *Laccaria* (Agaricomycetes, Basidiomycota) from Tibet (Xizang Autonomous Region, China). Mycoscience 54: 406–419. 10.1016/j.myc.2013.01.006

[B52] Wilson AW Hosaka K Mueller GM (2016) Evolution of ectomycorrhizas as a driver of diversification and biogeographic patterns in the model mycorrhizal mushroom genus *Laccaria*. The New Phytologist 213: 1862–1873. 10.1111/nph.14270 PMC5324586 28164331

[B53] Wilson AW May TW Mueller GM (2017) Biogeography of the ectomycorrhizal mushroom genus*Laccaria*. Biogeography of mycorrhizal symbiosis. Cham: Springer International Publishing 230: 273–297. 10.1007/978-3-319-56363-3_13

[B54] Wu F Zhou LW Yang ZL Bau T Li TH Dai YC (2019) Resource diversity of Chinese macrofungi: Edible, medicinal and poisonous species. Fungal Diversity 98: 1–76. 10.1007/s13225-019-00432-7

[B55] Zhang M Gao XL Mu LQ Deng WQ (2023) Morphology and molecular phylogeny reveal five new species of *Laccaria* (Hydnangiaceae, Agaricales) from southern China. Journal of Fungi 9: 1179. 10.3390/jof9121179 PMC10744585 38132780

[B56] Zhang SF Gui Y Zhu GS Shang NJ Li B Yang TJ Gong GL Huang WB Liu ZB (2024) *Laccaria aguizhouensis* sp. nov. (Agaricales, Basidiomycota) from Southwest China. New Zealand Journal of Botany 62: 138–150. 10.1080/0028825X.2024.2304754

