# Supplementary material for: ﻿Three new species and two new records of the genus Laccaria (Agaricales, Basidiomycota) from subtropical China based on morphological and multi-locus phylogenetic evidence
Source: MycoKeys. 2025 Oct 13;123:147–70. doi: 10.3897/mycokeys.123.156526 (PMC12538215; doi:10.3897/mycokeys.123.156526)
Supplement: Supplementary material 2 — Supplementary images [file mycokeys-123-147-s002.pdf]

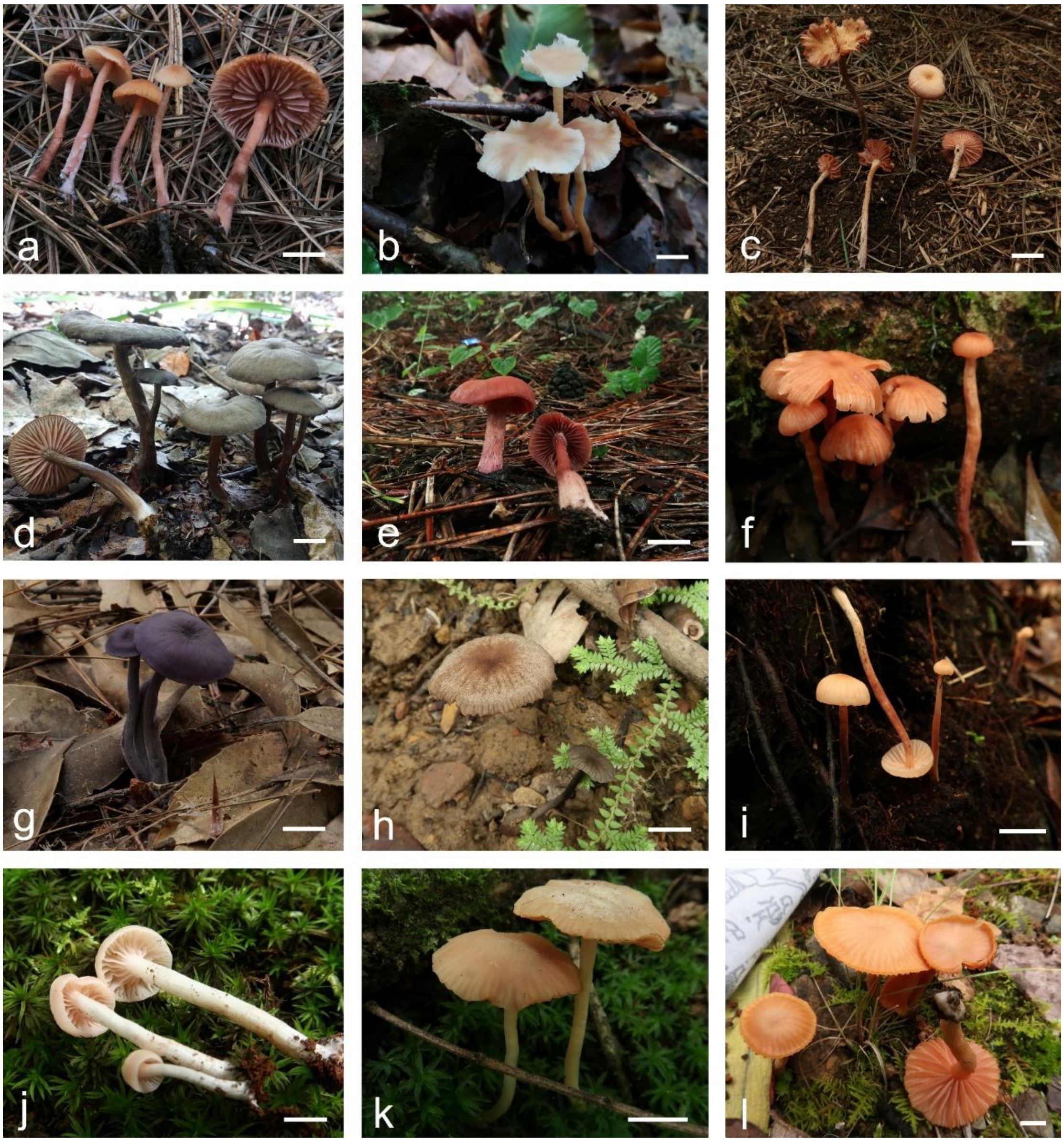

Figure S1 Basidiomata of *Laccaria alba* (**a** MHHNU 20007); *L. araneosa* (**b** MHHNU 34709); *L. aurantia* (**c** MHHNU 11885); *L. brunnea* (**d** MHHNU 9589); *L. bicolor* (**e** MHHNU 11595); *L. fagacicola* (**f** MHHNU 11978); *L. moshuijun* (**g** MHHNU 32931); *L. murina* (**h** MHHNU 10903); *L. rubroalba* (**i** MHHNU 11941); *L. stipalba* (**j** MHHNU 11315 and **k** MHHNU 12057); *L. acanthospora* (**l** MHHNU 12061) Bars = 1 cm.

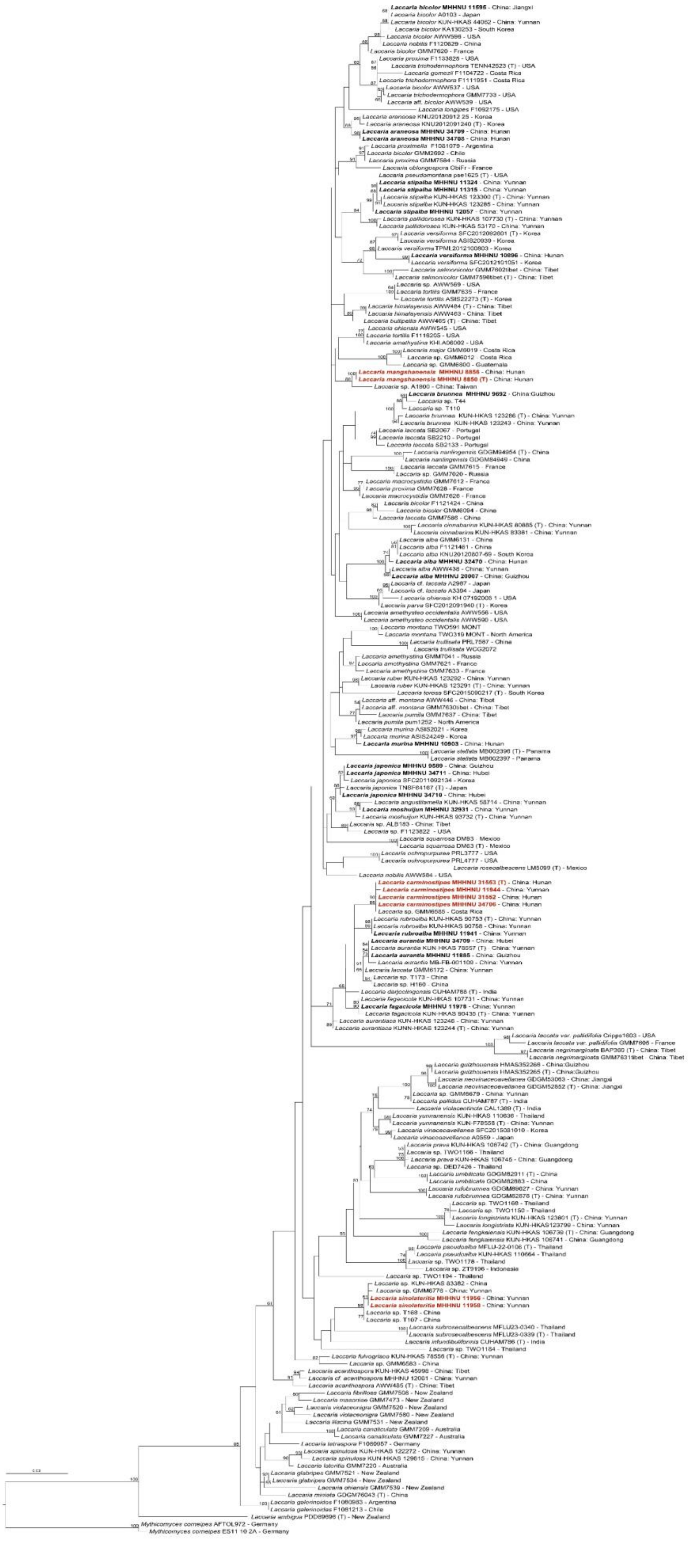

Figure S2 The maximum likelihood (ML) tree of *Laccaria* inferred from the ITS dataset. ML bootstrap values (BP) over 50% is shown at nodes. Newly generated sequences are highlighted in bold. GenBank accession numbers of sequences and their geographic origins are shown. Taxa marked with (T) represent type specimens.

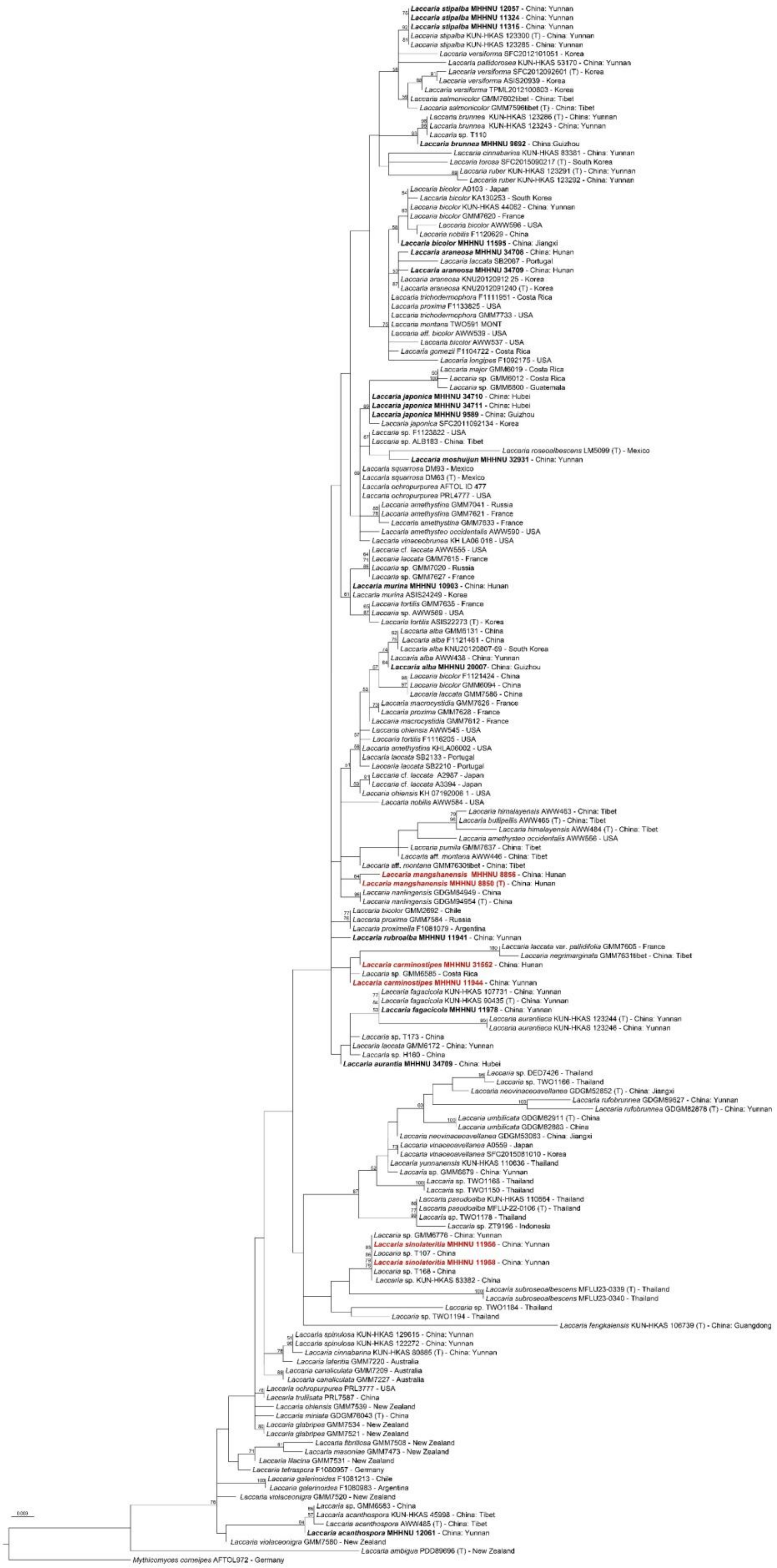
Laccaria stipalba MHHNU 12057 - China: Yunnan
Laccaria stipalba MHHNU 11324 - China: Yunnan
Laccaria stipalba MHHNU 11315 - China: Yunnan
Laccaria stipalba KUN-HKAS 123300 (T) - China: Yunnan
Laccaria stipalba KUN-HKAS 123285 - China: Yunnan
Laccaria versiforma SFC2012101051 - Korea
Laccaria pallidorosea KUN-HKAS 53170 - China: Yunnan
Laccaria versiforma SFC2012092601 (T) - Korea
Laccaria versiforma ASIS20939 - Korea
Laccaria versiforma TPML2012100803 - Korea
Laccaria salmonicolor GMM7602tibet - China: Tibet
Laccaria salmonicolor GMM7596tibet (T) - China: Tibet
Laccaria brunnea KUN-HKAS 123286 (T) - China: Yunnan
Laccaria brunnea KUN-HKAS 123243 - China: Yunnan
Laccaria sp. T110
Laccaria brunnea MHHNU 9692 - China:Guizhou
Laccaria cinnabarina KUN-HKAS 83381 - China: Yunnan
Laccaria torosa SFC2015090217 (T) - South Korea
Laccaria ruber KUN-HKAS 123291 (T) - China: Yunnan
Laccaria ruber KUN-HKAS 123292 - China: Yunnan
Laccaria bicolor A0103 - Japan
Laccaria bicolor KA130253 - South Korea
Laccaria bicolor KUN-HKAS 44062 - China: Yunnan
Laccaria bicolor GMM7620 - France
Laccaria bicolor AWW596 - USA
Laccaria nobilis F1120629 - China
Laccaria bicolor MHHNU 11595 - China: Jiangxi
Laccaria araneosa MHHNU 34708 - China: Hunan
Laccaria laccata SB2067 - Portugal
Laccaria araneosa MHHNU 34709 - China: Hunan
Laccaria araneosa KNU20120912 25 - Korea
Laccaria araneosa KNU2012091240 (T) - Korea
Laccaria trichodermophora F1111951 - Costa Rica
Laccaria proxima F1133825 - USA
Laccaria trichodermophora GMM7733 - USA
Laccaria montana TWO591 MONT
Laccaria aff. bicolor AWW539 - USA
Laccaria bicolor AWW537 - USA
Laccaria gomezii F1104722 - Costa Rica
Laccaria longipes F1092175 - USA
Laccaria major GMM6019 - Costa Rica
Laccaria sp. GMM6012 - Costa Rica
Laccaria sp. GMM6800 - Guatemala
Laccaria japonica MHHNU 34710 - China: Hubei
Laccaria japonica MHHNU 34711 - China: Hubei
Laccaria japonica MHHNU 9589 - China: Guizhou
Laccaria japonica SFC2011092134 - Korea
Laccaria sp. F1123822 - USA
Laccaria sp. ALB183 - China: Tibet
Laccaria roseoalbescens LM5099 (T) - Mexico
Laccaria moshuijun MHHNU 32931 - China: Yunnan
Laccaria squarrosa DM93 - Mexico
Laccaria squarrosa DM63 (T) - Mexico
Laccaria ochropurpurea AFTOL ID 477
Laccaria ochropurpurea PRL4777 - USA
Laccaria amethystina GMM7041 - Russia
Laccaria amethystina GMM7621 - France
Laccaria amethystina GMM7633 - France
Laccaria amethysteo occidentalis AWW590 - USA
Laccaria vinaceobrunea KH LA06 018 - USA
Laccaria cf. laccata AWW555 - USA
Laccaria laccata GMM7615 - France
Laccaria sp. GMM7020 - Russia
Laccaria sp. GMM7627 - France
Laccaria murina MHHNU 10903 - China: Hunan
Laccaria murina ASIS24249 - Korea
Laccaria tortilis GMM7635 - France
Laccaria sp. AWW569 - USA
Laccaria fortilis ASIS22273 (T) - Korea
Laccaria alba GMM6131 - China
Laccaria alba F1121461 - China
Laccaria alba KNU20120807-69 - South Korea
Laccaria alba AWW438 - China: Yunnan
Laccaria alba MHHNU 20007- China: Guizhou
Laccaria bicolor F1121424 - China
Laccaria bicolor GMM6094 - China
Laccaria laccata GMM7586 - China
Laccaria macrocystidia GMM7626 - France
Laccaria proxima GMM7628 - France
Laccaria macrocystidia GMM7612 - France
Laccaria ohiensis AWW545 - USA
Laccaria fortilis F1116205 - USA
Laccaria amethystina KHLA06002 - USA
Laccaria laccata SB2133 - Portugal
Laccaria laccata SB2210 - Portugal
Laccaria cf. laccata A2987 - Japan
Laccaria cf. laccata A3394 - Japan
Laccaria ohiensis KH 07192006 1 - USA
Laccaria nobilis AWW584 - USA
Laccaria himalayensis AWW463 - China: Tibet
Laccaria bullipellis AWW465 (T) - China: Tibet
Laccaria himalayensis AWW484 (T) - China: Tibet
Laccaria amethysteo occidentalis AWW556 - USA
Laccaria pumila GMM7637 - China: Tibet
Laccaria aff. montana AWW446 - China: Tibet
Laccaria aff. montana GMM7630tibet - China: Tibet
Laccaria mangshanensis MHHNU 8856 - China: Hunan
Laccaria mangshanensis MHHNU 8850 (T) - China: Hunan
Laccaria nanlingensis GDGM84949 - China
Laccaria nanlingensis GDGM94954 (T) - China
Laccaria bicolor GMM2692 - Chile
Laccaria proxima GMM7584 - Russia
Laccaria proximella F1081079 - Argentina
Laccaria rubroalba MHHNU 11941 - China: Yunnan
Laccaria laccata var. pallidifolia GMM7605 - France
Laccaria negrimarginata GMM7631tibet - China: Tibet
Laccaria carminostipes MHHNU 31552 - China: Hunan
Laccaria sp. GMM6585 - Costa Rica
Laccaria carminostipes MHHNU 11944 - China: Yunnan
Laccaria fagacicola KUN-HKAS 107731 - China: Yunnan
Laccaria fagacicola KUN-HKAS 90435 (T) - China: Yunnan
Laccaria fagacicola MHHNU 11978 - China: Yunnan
Laccaria aurantisca KUN-HKAS 123244 (T) - China: Yunnan
Laccaria aurantisca KUN-HKAS 123246 - China: Yunnan
Laccaria sp. T173 - China
Laccaria laccata GMM6172 - China: Yunnan
Laccaria sp. H160 - China
Laccaria aurantia MHHNU 34709 - China: Hubei
Laccaria sp. DED7426 - Thailand
Laccaria sp. TWO1166 - Thailand
Laccaria neovinaceoavellanea GDGM52852 (T) - China: Jiangxi
Laccaria rufobrunnea GDGM89627 - China: Yunnan
Laccaria rufobrunnea GDGM82878 (T) - China: Yunnan
Laccaria umbilicata GDGM82911 (T) - China
Laccaria umbilicata GDGM82883 - China
Laccaria neovinaceoavellanea GDGM53083 - China: Jiangxi
Laccaria vinaceoavellanea A0559 - Japan
Laccaria vinaceoavellanea SFC2015081010 - Korea
Laccaria yunnanensis KUN-HKAS 110636 - Thailand
Laccaria sp. GMM6879 - China: Yunnan
Laccaria sp. TWO1168 - Thailand
Laccaria sp. TWO1150 - Thailand
Laccaria pseudoalba KUN-HKAS 110664 - Thailand
Laccaria pseudoalba MFLU-22-0106 (T) - Thailand
Laccaria sp. TWO1178 - Thailand
Laccaria sp. ZT9196 - Indonesia
Laccaria sp. GMM6776 - China: Yunnan
Laccaria sinolateritia MHHNU 11956 - China: Yunnan
Laccaria sp. T107 - China
Laccaria sinolateritia MHHNU 11958 - China: Yunnan
Laccaria sp. T168 - China
Laccaria sp. KUN-HKAS 83382 - China
Laccaria subroseoalbescens MFLU23-0339 (T) - Thailand
Laccaria subroseoalbescens MFLU23-0340 - Thailand
Laccaria sp. TWO1184 - Thailand
Laccaria sp. TWO1194 - Thailand
Laccaria fengkaiensis KUN-HKAS 106739 (T) - China: Guangdong
Laccaria spinulosa KUN-HKAS 129615 - China: Yunnan
Laccaria spinulosa KUN-HKAS 122272 - China: Yunnan
Laccaria cinnabarina KUN-HKAS 80885 (T) - China: Yunnan
Laccaria lateritia GMM7220 - Australia
Laccaria canaliculata GMM7209 - Australia
Laccaria canaliculata GMM7227 - Australia
Laccaria ochropurpurea PRL3777 - USA
Laccaria trullisata PRL7587 - China
Laccaria ohiensis GMM7539 - New Zealand
Laccaria miniata GDGM76043 (T) - China
Laccaria glabripes GMM7534 - New Zealand
Laccaria glabripes GMM7521 - New Zealand
Laccaria fibrillosa GMM7508 - New Zealand
Laccaria masoniae GMM7473 - New Zealand
Laccaria lilacina GMM7531 - New Zealand
Laccaria tetraspora F1080957 - Germany
Laccaria galerinoides F1081213 - Chile
Laccaria galerinoides F1080983 - Argentina
Laccaria violaceonigra GMM7520 - New Zealand
Laccaria sp. GMM6583 - China
Laccaria acanthospora KUN-HKAS 45998 - China: Tibet
Laccaria acanthospora AWW485 (T) - China: Tibet
Laccaria acanthospora MHHNU 12061 - China: Yunnan
Laccaria violaceonigra GMM7580 - New Zealand
Laccaria ambigua PDD89696 (T) - New Zealand
Mythicomyces corneipes AFTOL972 - Germany
0.003

Figure S3 The maximum likelihood (ML) tree of *Laccaria* inferred from the LSU dataset. ML bootstrap values (BP) over 50% is shown at nodes. Newly generated sequences are highlighted in bold. GenBank accession numbers of sequences and their geographic origins are shown. Taxa marked with (T) represent type specimens.

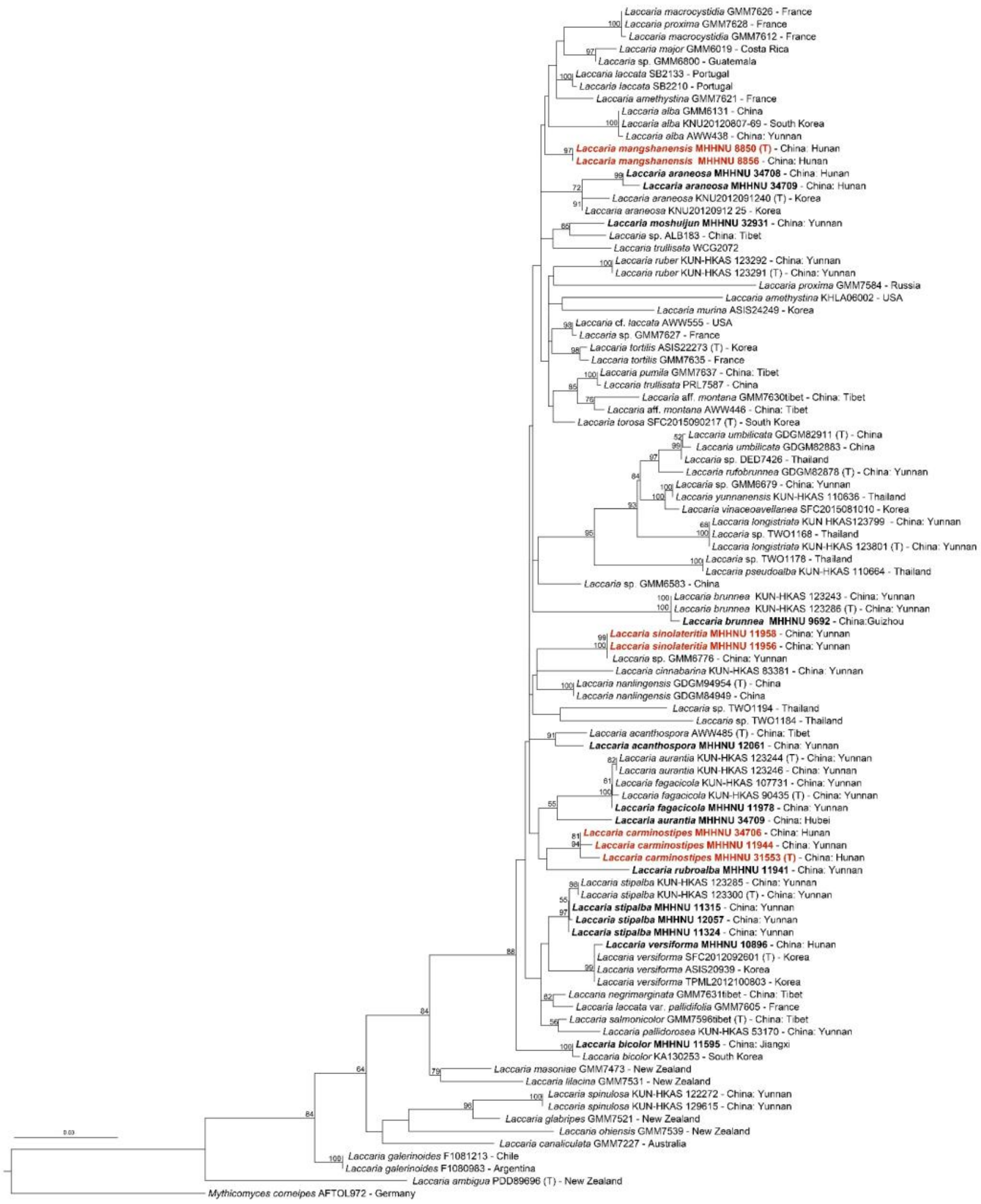

Figure S4 The maximum likelihood (ML) tree of *Laccaria* inferred from the *TEF1* dataset. ML bootstrap values (BP) over 50% is shown at nodes. Newly generated sequences are highlighted in bold. GenBank accession numbers of sequences and their geographic origins are shown. Taxa marked with (T) represent type specimens.

…

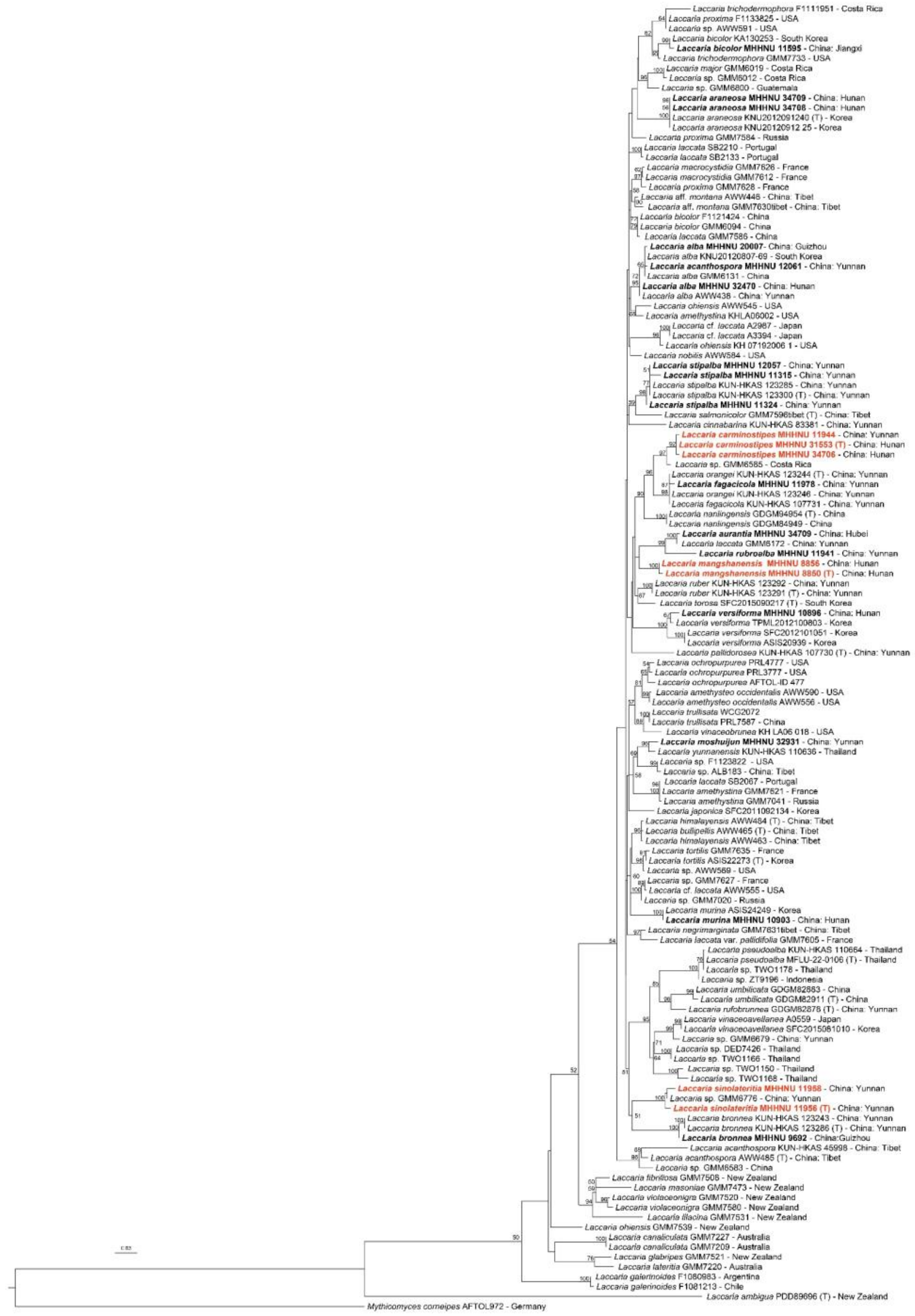

Figure S5 The maximum likelihood (ML) tree of *Laccaria* inferred from the *RPB2* dataset. ML bootstrap values (BP) over 50% is shown at nodes. Newly generated sequences are highlighted in bold. GenBank accession numbers of sequences and their geographic origins are shown. Taxa marked with (T) represent type specimens.
